# Quantification of chondroitin sulfate, hyaluronic acid and *N*-glycans in synovial fluid – A technical performance study

**DOI:** 10.1016/j.ocarto.2023.100380

**Published:** 2023-06-10

**Authors:** Elin Andersson, Emil Tykesson, L. Stefan Lohmander, Niclas G. Karlsson, Chunsheng Jin, Ekaterina Mirgorodskaya, Per Swärd, André Struglics

**Affiliations:** a Lund University, Faculty of Medicine, Department of Clinical Sciences Lund, Orthopaedics, Lund, Sweden; b Lund University, Faculty of Medicine, Department of Experimental Medical Science, Sweden; c Department of Life Science and Health, Faculty of Health Science, Oslo Metropolitan University, Oslo, Norway; d Proteomics Core Facility at Sahlgrenska Academy, University of Gothenburg, SE40530, Gothenburg, Sweden

**Keywords:** Chondroitin sulfate, *N*-Glycan, Synovial fluid, HPLC, Mass spectrometry

## Abstract

**Objective:**

To validate a quantitative high performance liquid chromatography (HPLC) assay for chondroitin sulfate (CS) and hyaluronic acid (HA) in synovial fluid, and to analyze glycan-patterns in patient samples.

**Design:**

Synovial fluid from osteoarthritis (OA, n ​= ​25) and knee-injury (n ​= ​13) patients, a synovial fluid pool (SF-control) and purified aggrecan, were chondroitinase digested and together with CS- and HA-standards fluorophore labelled prior to quantitative HPLC analysis. *N*-glycan profiles of synovial fluid and aggrecan were assessed by mass spectrometry.

**Results:**

Unsaturated uronic acid and sulfated-*N*-acetylgalactosamine (ΔUA-GalNAc4S and ΔUA-GalNAc6S) contributed to 95% of the total CS-signal in the SF-control sample. For HA and the CS variants in SF-control the intra- and inter-experiment coefficient of variation was between 3–12% and 11–19%, respectively; tenfold dilution gave recoveries between 74 and 122%, and biofluid stability test (room temperature storage and freeze-thaw cycles) showed recoveries between 81 and 140%. Synovial fluid concentrations of the CS variants ΔUA-GalNAc6S and ΔUA2S-GalNAc6S were three times higher in the recent injury group compared to the OA group, while HA was four times lower. Sixty-one different *N*-glycans were detected in the synovial fluid samples, but there were no differences in levels of *N*-glycan classes between patient groups. The CS-profile (levels of ΔUA-GalNAc4S and ΔUA-GalNAc6S) in synovial fluid resembled that of purified aggrecan from corresponding samples; the contribution to the *N*-glycan profile in synovial fluid from aggrecan was low.

**Conclusions:**

The HPLC-assay is suitable for analyzing CS variants and HA in synovial fluid samples, and the GAG-pattern differs between OA and recently knee injured subjects.

## 1 Introduction

Synovial fluid is a viscous liquid comprised of plasma transudate and various molecules synthesized by joint tissue cells. Its major functions are to lubricate the synovial joints and provide nutrients for the intra-articular structures. Proteoglycans, consisting of a protein core with attached glycosaminoglycan (GAG) side chains, are important components of the synovial fluid and extracellular matrix of articular cartilage. The GAGs consist of different types of repeating disaccharide units [1].

Aggrecan is the most abundant proteoglycan in articular cartilage, and its degradation has a central role in the pathogenesis of osteoarthritis (OA) [2]. The core protein of aggrecan has covalently attached *N*-linked (via Asn) and *O*-linked (via Ser or Thr) oligosaccharides. GAG chains on aggrecan are found as *O*-linked chondroitin sulfate (CS) and keratan sulfate (KS). *N*-linked oligosaccharides, herein named *N*-glycans, are composed of a variety of branched monosaccharides, mainly *N*-acetylglucosamine (GlcNAc), mannose, fucose, galactose and *N*-acetylneuraminic acid [3]. The CS chains are covalently attached via a linkage region to a Ser residue, where the main structure of the CS-chain is composed of disaccharide repeats of glucuronic acid (GlcA) and *N*-acetylgalactosamine (GalNAc) [4]. The articular cartilage KS chains (also called KS-II) are covalently attached via GalNAc to Ser or Thr residues, via a structure known as mucin core-2, on aggrecan and other proteoglycans. The main structure of the KS-chain consists of 3- and 6-sulfated disaccharide repeats of Gal and GlcNAc [5]. Due to the sulfation of the CS- and KS-chains, these GAGs attract water, giving the cartilage its load-bearing properties and ability to resist compression. Aggrecan forms aggregates through interaction with hyaluronic acid (HA), a high molecular weight non-sulfated GAG of repeated disaccharides of GlcA and GlcNAc, which together with lubricin aid joint lubrication [6,7].

The concentration of GAG in synovial fluid changes with different joint diseases, where the levels of HA and total GAG are decreased in arthritis patients compared with knee-healthy individuals [8,9]. The length and sulfation pattern of CS- and KS-chains change with age. With increasing age, the CS-chains become shorter and the KS-chains become longer, while on the CS-chains 6-sulfation of GalNAc increases and 4-sulfation decreases [2,10,11]. Depending on cleavage site and type of protease, the degradation of aggrecan is differently affected by the presence or absence of CS- and KS-chains [[12], [13], [14], [15]]. Extracellular proteins such as aggrecan may be primed with specific glycan-patterns which result in their degradation, and hence may play a role in the pathogenesis of OA and other joint diseases.

To better understand the role of glycans in the normal ageing and degradation of articular cartilage in the setting of OA, validated methods for detecting and quantifying GAG sulfation pattern and *N*-glycans in synovial fluid are needed. High performance liquid chromatography (HPLC) has been used for GAG assessments and mass spectrometry for *N*-glycan analysis [[16], [17], [18]]. However, a validation based on technical performance of these methods have to our knowledge not been published. In the present study, we aim to: (I) validate quantitative HPLC of CS and HA in synovial fluid samples; (II) as a proof of concept, examine the glycan-pattern of CS and HA in synovial fluid of OA and knee injury patients; (III) identify *N*-glycosylated proteins in synovial fluid and quantify *N*-glycans in the patient samples.

## 2 Materials and methods

### 2.1 Subjects and samples

From a cross-sectional convenience cohort, we selected patients with OA or knee injuries (Table 1), where the sample volume was sufficiently large for both HPLC analysis and *N-*glycan assessment. Thirteen samples were age-matched. The subjects had synovial fluid aspirated (without lavage), the samples were centrifuged at 3000×*g* for 10 min at 4 °C, and the cell/cell-debris free synovial fluids were stored at −80 °C. All knee-injured subjects (n = 13) had hemarthrosis with the following magnetic resonance imaging (MRI) based main diagnosis: anterior cruciate ligament rupture (n = 10), meniscus injury (n = 5) or patella luxation (n = 1). Aspiration of the injured knees was done between 0 and 5 days after knee trauma, herein called recent knee injury. The OA group (n = 25) included subjects with early to late-stage OA (based on x-ray examination). For this study we also used a synovial fluid control sample (SF-control); from a pool of synovial fluid from seven knee-injured subjects and a CS quality control sample (CS-QC; Sigma no. C4384) which was solubilized in Milli-Q water (10 μg/μl). The study was approved by the regional ethical review board (Lund, Sweden). The samples have been used in previous investigations [[19], [20], [21], [22], [23], [24]].

**Table 1:** Characteristics and demographics of subject groups.

Main diagnostic groups	Sub-groups of OA	N (% women)	Age in years, median (range)	Differences in age, p-values	Differences in sex, p-values
				Recent injury vs	Recent injury vs
Recent injury		13 (46.2)	41.0 (36.2–64.4)	–	–
Osteoarthritis		25 (40.0)	61.3 (35.7–86.3)	**<0.001**	0.715
	*Age-matched OA*	13 (23.1)	48.0 (35.7–63.6)	0.099	0.216

Within the recent knee injury group, women (median age ​= ​39.6, n ​= ​6) were younger (P ​= ​0.022) than men (median age ​= ​42.3, n ​= ​7); within the osteoarthritis (OA) group, women (median age ​= ​67.8, n ​= ​10) were older (P ​= ​0.004) than men (median age ​= ​56.0, n ​= ​15). There was a difference (using alpha value = 0.05; P-value marked in bold) in age between the OA and recent knee injury groups (median years, 61 vs 41); this difference was abolished between age-matched OA and recent knee injury groups (median years, 48 vs 41).

### 2.2 Pretreatment of synovial fluid for HPLC analysis

#### 2.2.1 Chondroitinase digestion

Synovial fluid samples were digested with chondroitinase ABC (Sigma no. C2905), using 5 mIU (milli-international units) per μg sulfated GAG (sGAG; assessed by Alcian blue: see below), or 1 mIU per μl synovial fluid, at 37 °C for 16 h in 50 mM ammonium acetate buffer pH 8. CS-QC were digested in a similar way using 5 mIU chondroitinase ABC per μg dry weight CS.

#### 2.2.2 Purification of aggrecan by boiling

Boiling is a simple and quick procedure to purify aggrecan [25]. Synovial fluid samples were after chondroitinase digestion boiled at 100 °C for 10 min and compared to treatment without boiling; both types of samples were centrifuged at 20 000×*g* for 5 min at room temperature (to remove non-aggrecan protein pellets), thereafter the supernatants containing aggrecan were collected.

#### 2.2.3 Hyaluronidase digestion

Synovial fluid samples were treated with hyaluronidase: 0.03 TRU (15 ng enzyme) hyaluronidase (*Streptomyces hyalurolyticus*; Seikagaku no.100740) per μg sGAG at 60 °C for 3 h in 50 mM ammonium-acetate buffer pH 6.0. Samples not treated with hyaluronidase were meanwhile stored at 4 °C.

#### 2.2.4 Filtration of synovial fluid

Synovial fluid samples were filtrated using 30 kDa cut-off filter (500-μl Amicon no. UFC 503024; according to Amicon user guide) and the filtrates were collected.

### 2.3 Disaccharide analysis

Chondroitinase digested samples and CS- and HA-standards (a mixture of nine standards; see below) were labelled with 2-aminoacridone (AMAC) and analyzed by HPLC as previously described [26]. Briefly, see workflow (Fig. S2): dried (by speed vac) samples (0.01–2.7 μg sGAG) and a standard mix (100 ng per standard) were fluorescently labelled by reductive amination using 10 μl of 20 mM AMAC (Sigma-Aldrich no. 06627) in acetic acid/dimethyl sulfoxide (15/85, v/v, pH 4–5). The samples were then incubated for 20 min at room temperature in darkness, followed by addition of 10 μl of 1 M cyanoborohydride and incubation at 37 °C overnight with 600 rpm shaking (Eppendorf Thermomixer C). AMAC-labelled synovial fluid samples, CS-QC and standards were analyzed using a quantitative HPLC assay. The following CS and HA disaccharide standards from Iduron were used (cat. no. CD001–CD008 and HA02): unsaturated uronic acid (ΔUA)-GalNAc, ΔUA-GalNAc4S, ΔUA-GalNAc6S, ΔUA-GalNAc4S6S, ΔUA2S-GalNAc4S, ΔUA2S-GalNAc6S, ΔUA2S-GalNAc4S6S, ΔUA2S-GalNAc and HA (ΔUA-GlcNAc). Typical HPLC-elution profiles are shown in Fig. S3.

### 2.4 *N*-glycan assessment with mass spectrometry

*N*-glycans were analyzed by mass spectrometry (MS). Briefly: 30 μl synovial fluid were reduced with dithiothreitol (DTT) and alkylated with iodoacetamide (IAA). 10 mU of Peptide: *N*-glycosidase F (PNGase F) was added, and the sample was incubated at 37 °C overnight. Released *N*-glycans were purified, reduced, desalted and analyzed by liquid chromatography-MS/MS coupled with a column packed with 5 μm porous graphite particles (Hypercarb, Thermo-Hypersil). The frequency of the *N*-glycans in the synovial fluid samples were calculated as the area under the curve (AUC) of each *N*-glycan structure divided by the total AUC expressed as a percentage. For a more detailed description of *N*-glycan assessment, see Supplementary Methods.

### 2.5 Global analysis of *N*-glycosylated proteins in synovial fluid

SF-control sample (1 mg) was reduced with DTT and alkylated with IAA. The sample was digested (at 37 °C) with Pierce MS grade trypsin, first with 10 μg for 4 h followed by an extra addition of 10 μg trypsin and an overnight incubation. The tryptic peptides were purified using Pierce peptide desalting spin columns followed by glycopeptide enrichment using in-house prepared hydrophilic interaction liquid chromatography (HILIC) spin columns. Both HILIC flow-through and eluate were collected and used for further proteomic and glycoproteomic analysis. HILIC flow-through fraction was further fractionated by basic reversed-phase chromatography. The HILIC eluate (enriched in glycopeptides) and the HILIC flow-through fractions were reconstituted in a solution of acetonitrile/formic acid, and thereafter NanoLC-MS was performed on QExactive HF mass spectrometer interfaced with an Easy-nLC1200 liquid chromatography system. The data was analyzed using Proteome Discoverer version 2.4 (Thermo Fisher Scientific): the data from HILIC flow-through fractions were matched against Swiss-Prot *Homo sapiens* database, using the Mascot search engine v. 2.5.1 (Matrix Science, London, UK); the data from HILIC enriched preparation were analyzed with Byonic (Protein Metrics) as search engines. The suggested glycosylated peptide identifications were evaluated based on the number of glycoforms per each site, number of peptide spectrum matches per glycoform (calculated as sum of all peptide spectrum matches in four independent injection) and the retention time window for the observed glycoforms with the same peptide core. For a more detailed description of global analysis of *N*-glycosylated proteins in synovial fluid, see Supplementary Methods.

### 2.6 Aggrecan purification from synovial fluid

Aggrecan was purified from synovial fluid samples (four recently knee-injured subjects and from SF-control), using density centrifugation (D1 mini prep) as described [25].

### 2.7 Analysis of sGAG

Analysis of sGAG, measured by Alcian blue precipitation (detecting CS and KS but not HA), was conducted as described previously [27,28].

### 2.8 Statistics

Based on test for normality (Shapiro-Wilk, histogram and Q-Q plots), the CS, HA and sGAG data were not normally distributed; therefore, non-parametric analyses were used for group comparisons (Kruskal-Wallis, Mann-Whitney Exact sig. 2-tailed and Wilcoxon signed-rank test) and correlation analyses (Spearman's rank order). Differences between OA and knee injury groups were analyzed by Student's t-test (for age) and Chi square (for sex). Age differences between men and women within the OA group were analyzed by Mann-Whitney test. If not otherwise specified, expressions such as “higher” and “lower” in the text are based on statistically significant differences. The level of significance was set at p < 0.05, and analyses were performed using the statistical software platform IBM SPSS Statistics 26.0. No adjustments for multiple comparisons were made.

## 3 Results

### 3.1 Different treatments of synovial fluid for the HPLC-assay

We evaluated the effect of CS disaccharide quantification pretreating synovial fluid with denaturation (heat to precipitate non–CS–containing proteins), hyaluronidase treatment and filtration. Results from the different treatments of synovial fluid (see Fig. S1) are presented in Table 2. The treatments were done in true replicates (here duplicates); i.e., as for treatment A1, two aliquots of the SF-control sample were separately treated with chondroitinase ABC, labelled and injected into the HPLC.

**Table 2:** Prior to labelling of CS and HA and injection into HLPC, synovial fluid control sample was processed according to different treatment conditions (A-D; Fig. S1). Duplicates per treatment (0.14 ​μl synovial fluid injected per treatment) were used, and the data is expressed as % of signal of mean values in relation to treatment A1 (only chondroitinase ABC digestion). Coefficients of variation (CV) for duplicates are shown as text below.

Treatments	ΔUA-GalNAc4S	ΔUA-GalNAc6S	ΔUA-GalNAc4S6S	ΔUA-GlcNAc (HA)	Total CS
A2	151.9	144.0	109.1	135.9	146.0
B1	104.4	109.7	81.6	100.4	109.7
B2	145.3	146.0	85.8	129.0	146.7
C1	139.3	131.8	111.2	121.4	131.9
C2	126.4	133.3	88.7	114.6	134.0
D1	128.0	129.3	121.8	113.7	128.1
D2	125.6	136.8	82.6	110.6	136.8

Treatments (CV span for duplicates): A1, chondroitinase (6.9–39.1%); A2, chondroitinase ​+ ​filtration (0.1–4.7%); B1, chondroitinase ​+ ​hyaluronidase (0.9–19.8%); B2, chondroitinase ​+ ​hyaluronidase ​+ ​filtration (2.6–7.6%); C1, chondroitinase ​+ ​boiling (3.4–13.3%); C2, chondroitinase ​+ ​boiling ​+ ​filtrations (0.3–7.1%); D1, chondroitinase ​+ ​boiling ​+ ​hyaluronidase (0.8–1.9%); D2, chondroitinase ​+ ​boiling ​+ ​hyaluronidase ​+ ​filtrations (1.9–5.0%). GalNAc = *N*-acetylgalactosamine, GlcNAc = *N*-acetylglucosamine, HA ​= ​hyaluronic acid, HPLC ​= ​high performance liquid chromatography, Total CS ​= ​sum of signal from all detected chondroitin sulfate disaccharides measured by the HPLC assay (i.e., ΔUA-GalNAc4S, ΔUA-GalNAc6S, ΔUA-GalNAc4S6S and ΔUA2S-GalNAc6S), ΔUA ​= ​unsaturated uronic acid, 2S ​= ​sulfation on carbon atom 2 of UA, 4S6S ​= ​sulfation on carbon atoms 4 and 6 of GalNAc.

We found that there was no apparent difference using removal of excess proteins by denaturation of synovial fluid (heating by boiling; a purification method for aggrecan) compared to no heating, since there was no major change in the signal of disaccharides generated from CS or HA (Table 2, Table S1). Lowering the synovial fluid injection volume five times (from 0.14 to 0.028 μL) resulted in not detecting the low abundant CS derived ΔUA-GalNAc4S6S (Table S1). Even though some of the treatments generated equal or higher HPLC signals (110–168%; Table 2) compared to the simplest treatment (A1 = only chondroitinase ABC digestion), we chose to continue with the A1 treatment for the rest of the study using an injection volume of 0.14 μL synovial fluid. This simple treatment is advantageous when screening patient synovial fluid samples and it minimizes the variations between samples, compared to when more complex treatments are used (i.e., treatments A2, B, C and D; Fig. S1).

**Fig. 1:**
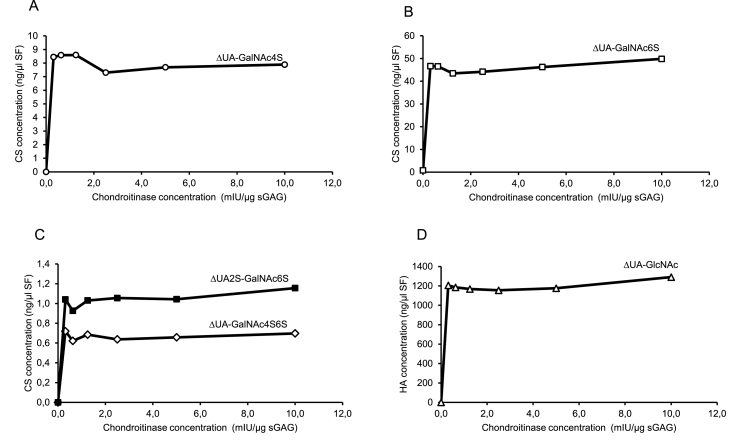
SF-control samples were digested with increasing amount of chondroitinase ABC for 16 ​h at 37 ​°C and injected to high performance liquid chromatography. Concentration based on received signal, expressed as ng chondroitin sulfate (CS) or hyaluronic acid (HA) per μl synovial fluid (SF), as an effect of different chondroitinase ABC concentrations are presented as mean values from duplicates. (A) unsaturated uronic acid (ΔUA)-*N*-acetylgalactosamine with sulfation on carbon atom 4 (GalNAc4S); (B) ΔUA-GalNAc6S; (C) ΔUA2S-GalNAc6S and ΔUA-GalNAc4S6S; (D) ΔUA-*N*-acetylglucosamine (GlcNAc), i.e., HA. sGAG ​= ​sulfated glycosaminoglycan (assessed by Alcian Blue assay), 4S6S ​= ​sulfation on carbon atoms 4 and 6 of GalNAc.

In another set of experiments, synovial fluid samples were digested with varying concentrations of chondroitinase ABC. As shown in Fig. 1, our default concentration of 5 mIU chondroitinase ABC per μg sGAG gave equal CS- and HA-signals as compared to higher enzyme concentrations. Therefore, we chose to continue with the default concentration for the rest of the study.

### 3.2 Technical performance of the CS and HA HPLC-assay

Since ΔUA-GalNAc, ΔUA2S-GalNAc and ΔUA2S-GalNAc4S were detected in none and ΔUA2S-GalNAc4S6S were only detected in two of the 38 subject synovial fluid samples, we did not investigate the technical performance of these disaccharides (Table 3). The signal area of ΔUA-GalNAc4S and ΔUA-GalNAc6S together represented 95% of the total CS (i.e., the sum of signals from all detected CS disaccharides measured by the HPLC assay) in the SF-control sample. These disaccharides also displayed low intra experiment CV values (mean CV between 3 and 7%) for SF-control and patient synovial fluid samples (Table 3). The inter experiment CV of the SF-control sample was between 11 and 19% for the CS variants and HA. For the CS-QC sample, the abundant CS variants ΔUA-GalNAc4S, ΔUA-GalNAc6S and ΔUA2S-GalNAc6S displayed intra- and inter-experimental CVs of 2.5–3.6% and 3.3–4.4%, respectively; the intra experiment CV of the CS-standards were 0.2–1.3%, and 7% for the HA-standard (Table 3).

**Table 3:** Technical performance of CS- and HA-HPLC assay.

Study						
*SF patients*^f^		ΔUA-GalNAc4S	ΔUA-GalNAc6S	ΔUA-GalNAc4S6S	ΔUA2S-GalNAc6S	ΔUA-GlcNAc (HA)
	N with data (%)	37 (97)	38 (100)	20 (53)	26 (68)	38 (100)
	Proportion (in %) to the standards, mean (min, max)	59 (20, 198)	312 (49, 1093)	23 (2, 68)	8 (3, 18)	6158 (333, 18 ​618)
	Concentration range; min, max	79.6, 586,7 ​ng/ml	197.5, 4372.8 ​ng/ml	22.8, 244.1 ​ng/ml	12.4, 73.0 ​ng/ml	8.7, 74.5 ​μg/ml
	aIntra experiment CV%:					
	Mean (min, max)	7.1 (0.3, 21.8)	3.9 (0.4, 10.4)	12.9 (0.4, 32.4)	12.4 (0.1, 32.4)	1.2 (0.7, 1.8)
	N in duplicates	31	32	19	24	32
Assay controls and standards						
*SF control*		ΔUA-GalNAc4S	ΔUA-GalNAc6S	ΔUA-GalNAc4S6S	ΔUA2S-GalNAc6S	HA
	bProportion of total CS-signal in sample (%)	11.0	84.1	0.9	1,8	Na
	Intra experiment CV %; mean (N ​= ​5 repeats)	4.4	3.3	12.1	3.4	3.9
	Inter experiment CV %; (N ​= ​3–5 experiments)	18.0	15.0	12.2	11.0	18.5
	Dilution recovery in % (% signal of standards):					
	cOriginal dilution	100 (59.7)	100 (345.8)	100 (4.3)	100 (6.5)	100 (4513)
	1:1.33	106 (47.5)	98 (253.0)	83 (2.7)	103 (5.0)	104 (3529)
	1:2	146 (43.4)	105 (182.4)	71 (1.5)	90 (2.9)	109 (2450)
	1:4	102 (15.3)	106 (91.5)	67 (0.7)	107 (1.8)	129 (1456)
	1:10	74 (4.4)	101 (35.0)	81 (0.3)	116 (0.8)	122 (550)
	1:20	131 (3.9)	125 (21.7)	*No signal*	*No signal*	154 (347)
	1:40	69 (1.0)	116 (10.0)	*No signal*	*No signal*	162 (183)
	1:100	*No signal*	159 (5.5)	*No signal*	*No signal*	252 (114)
	Stability, relative units (control/treatment) in %:					
	Storage 19 ​h at room temperature	105	113	140	111	104
	15 freeze/thaw cycles	81	94	116	96	86
*CS-QC*						
	dProportion of total HA ​+ ​CS-signal in sample (%)	33.4	36.5	0.8	24.6	1.4
	eIntra experiment CV %; mean (N ​= ​10 or 12 repeats)	2.5	3.9	7.9	3.6	9.5
	Inter experiment CV %; (N ​= ​5 or 6 experiments)	4.4	3.5	22.1	3.3	31.7
	Stability, relative units in % (control/treatment):					
	Stored 19 ​h at room temperature	103	108	105	106	123
	15 freeze/thaw cycles	96	101	103	99	102
g*CS- and HA-standards*						
	Intra experiment CV %; (N ​= ​4 or 5 repeats)	0.2	0.5	1.3	0.7	7.0

CV ​= ​coefficient of variation, CS ​= ​chondroitin sulfate, GalNAc = *N*-acetylgalactosamine, GlcNAc = *N*-acetylglucosamine, Na ​= ​not applicable, ΔUA ​= ​unsaturated uronic acid.

a From one experiment; chondroitinase digestion of samples and AMAC labelling of samples and standards at the same occasion, and quantification of these within one HPLC run.

b The synovial fluid (SF) control sample also contained a 6th peak that might correspond to ΔUA2S-GalNAc4S constituting to 2.1% of total CS-signal.

c The original dilution (set to 100% recovery) corresponds to 0.14 ​μl injected synovial fluid.

d The CS-QC sample also contained ΔUA2S-GalNAc4S and ΔUA-GalNAc corresponding to 0.8 and 2.9%, respectively.

e Duplicates per experiment using five or six experiments for calculation of mean CV.

f 0.14 ​μl synovial fluid was injected (dilution 1:50) into the HPLC.

g The standard mix also contain ΔUA-GalNAc, ΔUA2S-GalNAc, ΔUA2S-GalNAc4S6S and ΔUA2S-GalNAc4S which had intra experiment CV ​= ​0.8, 0.6, 3.1 and 1.0%, respectively. The CV of hyaluronic acid (HA) was based on signal from UV-VIS values while the CV for the CS-standards was based on emission values.

Tenfold dilution of the SF-control sample (corresponding to 14 nL synovial fluid) gave acceptable recoveries (74–122% of signal vs undiluted sample), while further dilution displayed loss of signal and worse recoveries. The stability tests (room temperature or freeze-thaw cycles treatments) of the SF-control sample displayed recoveries of 81–140%. Similar stability recoveries were found for the CS-QC sample (Table 3).

### 3.3 Concentration of CS variants and HA in synovial fluid of patients

We found that the concentrations of CS variants ΔUA-GalNAc6S and ΔUA2S-GalNAc6S were each approximately three times *higher* for the recent knee injury group compared to the levels found in the age-matched OA group. The concentrations of total CS and sGAG (sulfated GAG, assessed by Alcian Blue) were also (two and three times, respectively) *higher* for the recent knee injury group compared to the levels found in the age-matched OA group (Table 4). On the other hand, the levels of HA and HA/total CS ratio were four and nine times, respectively, *lower* for the recent knee injury group compared to the levels found in the age-matched OA group. There was no difference in concentrations of CS variants and HA between the sexes in the OA group, nor in the recent knee injury group (data not shown).

**Table 4:** Concentrations of CS variants and HA and sGAG in synovial fluid from different patient groups. Normalization (injury divided by OA) is based on median. Statistical significance (p ​< ​0.05) is marked in bold.

ΔUA-GalNAc4S							
	Groups	Median (min, max), ng/ml	n	OA vs injury		Injury vs OA age matched	
				Norm	P values	Norm	P values
	OA	168.4 (79.6, 792.2)	24	1	–	–	–
	OA age-matched	169.1 (79.6, 426.9)	13	–	–	1	–
	Recent knee injury	344.4 (86.2, 586.7)	13	2.05	0.067	2.04	0.113
ΔUA-GalNAc6S							
	Groups	Median (min, max), ng/ml	n	OA vs injury		Injury vs OA age matched	
				Norm	P values	Norm	P values
	OA	578.5 (197.5, 4053.7)	25	1	–	–	–
	OA age-matched	725.3 (197.5, 4053.7)	13	–	–	1	–
	Recent knee injury	1925.3 (485.1, 4372.8)	13	3.33	**0.001**	2.65	**0.016**
ΔUA-GalNAc4S6S							
	Groups	Median (min, max), ng/ml	n	OA vs injury		Injury vs OA age matched	
				Norm	P values	Norm	P values
	OA	75.6 (22.8, 273.4)	14	1	–	–	–
	OA age-matched	44.1 (22.8, 244.1)	5	–	–	1	–
	Recent knee injury	44.9 (8.5, 138.1)	6	0.59	0.062	1.02	0.931
ΔUA2S-GalNAc6S							
	Groups	Median (min, max), ng/ml	n	OA vs injury		Injury vs OA age matched	
				Norm	P values	Norm	P values
	OA	24.4 (12.4, 45.1)	16	1	–	–	–
	OA age-matched	18.2 (12.4, 38.9)	6	–	–	1	–
	Recent knee injury	49.0 (16.3, 72.98)	10	2.01	**0.022**	2.69	**0.031**
Total CS^a^							
	Groups	Median (min, max), ng/ml	n	OA vs injury		Injury vs OA age matched	
				Norm	P values	Norm	P values
	OA	893.3 (277.1, 4446.3)	25	1	–	–	–
	OA age-matched	941.6 (277.1, 4446.3)	13	–	–	1	–
	Recent knee injury	2278.1 (617.4, 4851.1)	13	2.55	**0.005**	2.42	**0.029**
sGAG							
	Groups	Median (min, max), μg/ml	n	OA vs injury		Injury vs OA age matched	
				Norm	P values	Norm	P values
	OA	42.4 (8.0, 201.0)	24	1	–	–	–
	OA age-matched	58.0 (17.0, 201.0)	13	–	–	1	–
	Recent knee injury	180.2 (23.68, 546.9)	12	4.25	**<0.001**	3.11	**0.026**
ΔUA-GlcNAc (HA)							
	Groups	Median (min, max), μg/ml	n	OA vs injury		Injury vs OA age matched	
				Norm	P values	Norm	P values
	OA	28.7 (8.7, 74.5)	25	1	–	–	–
	OA age-matched	28.9 (18.7, 74.5)	13	–	–	1	–
	Recent knee injury	7.8 (1.3, 38.0)	13	0.27	**<0.001**	0.27	**<0.001**
HA/total-CS							
	Groups	Median (min, max)	n	OA vs injury		Injury vs OA age matched	
				Norm	P values	Norm	P values
	OA	31.7 (2.3, 116.5)	25	1	–	–	–
	OA age-matched	30.9 (2.3, 116.5)	13	–	–	1	–
	Recent knee injury	3.4 (1.2, 34.2)	13	0.11	**<0.001**	0.11	**<0.001**

a Total CS is equal to the sum of signals from all detected chondroitin sulfate disaccharides measured by the HPLC assay (i.e., ΔUA-GalNAc4S, ΔUA-GalNAc6S, ΔUA-GalNAc4S6S and ΔUA2S-GalNAc6S). sGAG ​= ​sulfated glycosaminoglycans assessed by Alcian blue assay, GalNAc = N-acetylgalactosamine, GlcNAc = N-acetylglucosamine, HA ​= ​hyaluronic acid, ΔUA ​= ​unsaturated uronic acid, 2S ​= ​sulfation on carbon atom 2 of UA, 4S6S ​= ​sulfation on carbon atoms 4 and 6 of GalNAc.

### 3.4 Correlation between CS variants and HA, sGAG and age

For these correlation assessments, we merged the data from the two patient groups (OA plus recent injury, total n = 38; Table 5). Synovial fluid concentrations of HA and ΔUA-GalNAc4S6S correlated positively with age, while ΔUA-GalNAc6S and sGAG correlated negatively with age. Consistent with this, the concentration of HA correlated negatively with ΔUA-GalNAc6S, total CS and sGAG, and positively with ΔUA-GalNAc4S6S. The CS variants ΔUA-GalNAc4S, ΔUA-GalNAc6S and ΔUA2S-GalNAc6S correlated positively with ΔUA-GalNAc4S6S, while ΔUA-GalNAc6S correlated negatively with ΔUA-GalNAc4S6S; sGAG correlated positively with total CS (Table 5).

**Table 5:** Non-parametric correlation using all samples (OA and recent knee injury; total n ​= ​38).

		ΔUA-GalNAc4S	ΔUA-GalNAc6S	ΔUA-GalNAc4S6S	ΔUA2S-GalNAc6S	Total CS	sGAG	ΔUA-GlcNAc (HA)
Age	r_S_	−0.007	**−0.333**	**0.532**	−0.015	−0.202	**−0.528**	**0.420**
	P	0.966	**0.041**	**0.016**	0.943	0.224	**0.001**	**0.009**
	N	37	38	20	26	38	36	38
ΔUA-GalNAc4S	r_S_		**0.743**	−0.189	**0.829**	**0.824**	**0.599**	−0.228
	P		**< 0.001**	0.437	**< 0.001**	**< 0.001**	**< 0.001**	0.175
	N		37	19	25	37	36	37
ΔUA-GalNAc6S	r_S_			**−0.471**	**0.557**	**0.968**	**0.890**	**−0.462**
	P			**0.036**	**0.003**	**< 0.001**	**< 0.001**	**0.003**
	N			20	26	38	36	38
ΔUA-GalNAc4S6S	r_S_				0.245	−0.149	**−0.674**	**0.868**
	P				0.298	0.531	**0.002**	**< 0.001**
	N				20	20	18	20
ΔUA2S-GalNAc6S	r_S_					**0.713**	**0.523**	−0.093
	P					**< 0.001**	**0.009**	0.653
	N					26	24	26
Total CS^a^	r_S_						**0.818**	**−0.325**
	P						**< 0.001**	**0.046**
	N						36	38
sGAG	r_S_							**−0.472**
	P							**0.004**
	N							36

a Total CS is equal to the sum of signals from all detected chondroitin sulfate disaccharides measured by the HPLC assay (i.e., ΔUA-GalNAc4S, ΔUA-GalNAc6S, ΔUA-GalNAc4S6S and ΔUA2S-GalNAc6S). sGAG ​= ​sulfated glycosaminoglycans assessed by the Alcian blue assay, GalNAc = *N*-acetylgalactosamine, GlcNAc = *N*-acetylglucosamine, HA ​= ​hyaluronic acid, OA ​= ​osteoarthritis, ΔUA ​= ​unsaturated uronic acid, 2S ​= ​sulfation on carbon atom 2 of UA, 4S6S ​= ​sulfation on carbon atoms 4 and 6 of GalNAc.

### 3.5 CS profile in aggrecan and synovial fluid

**Fig. 2:**
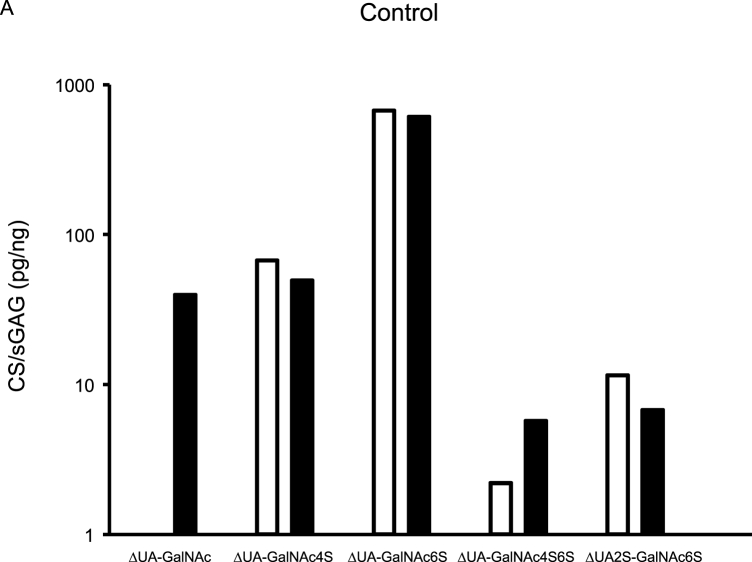

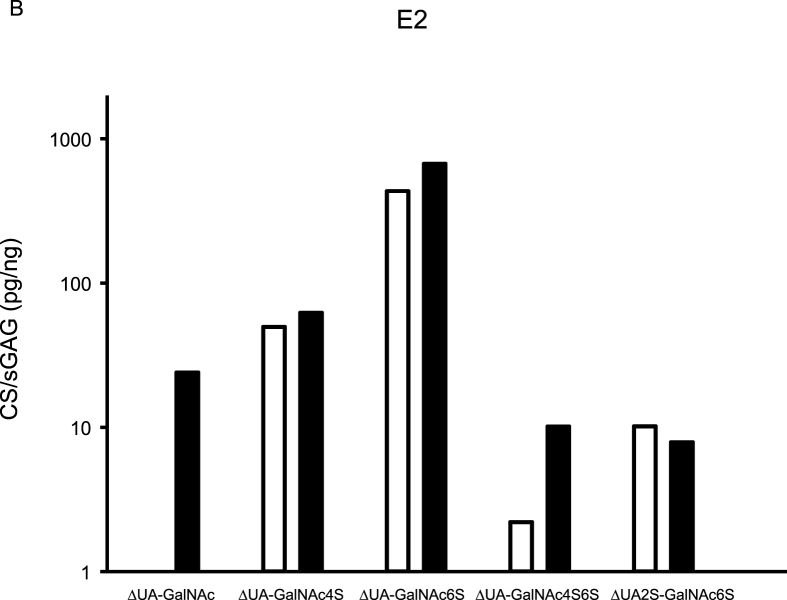

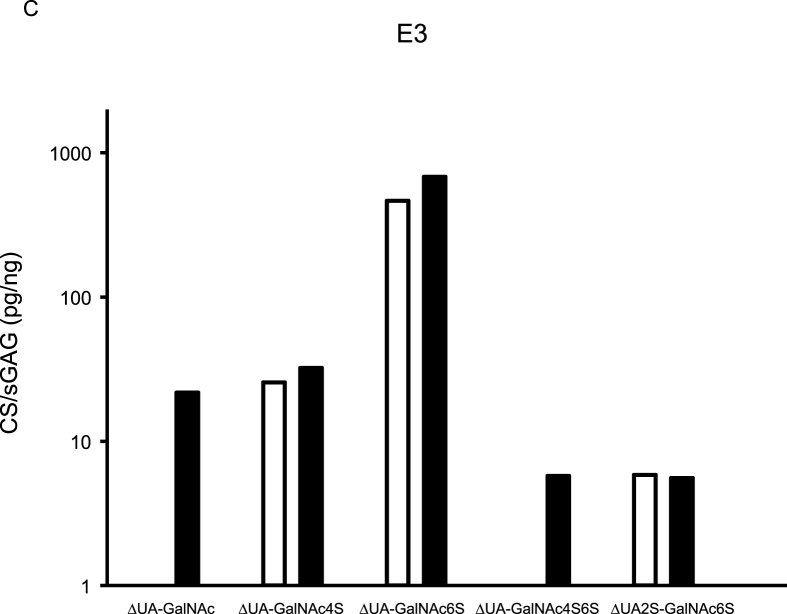

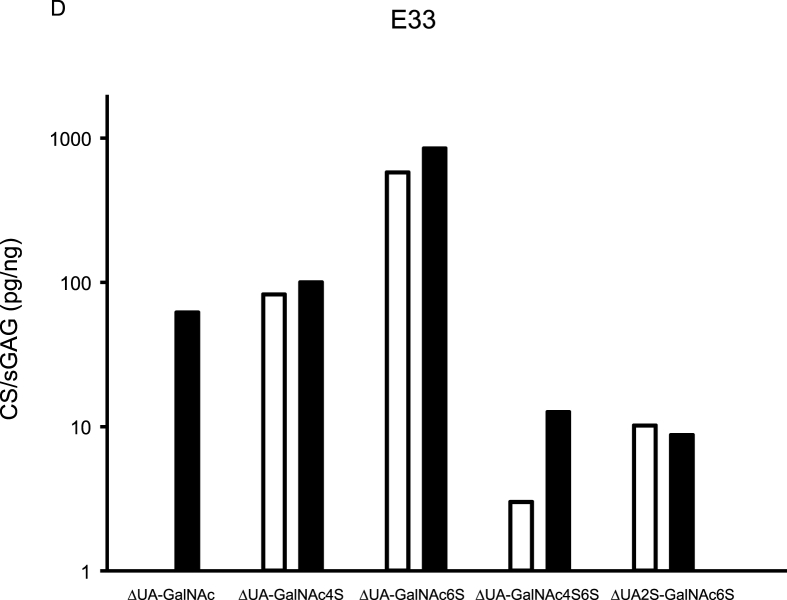

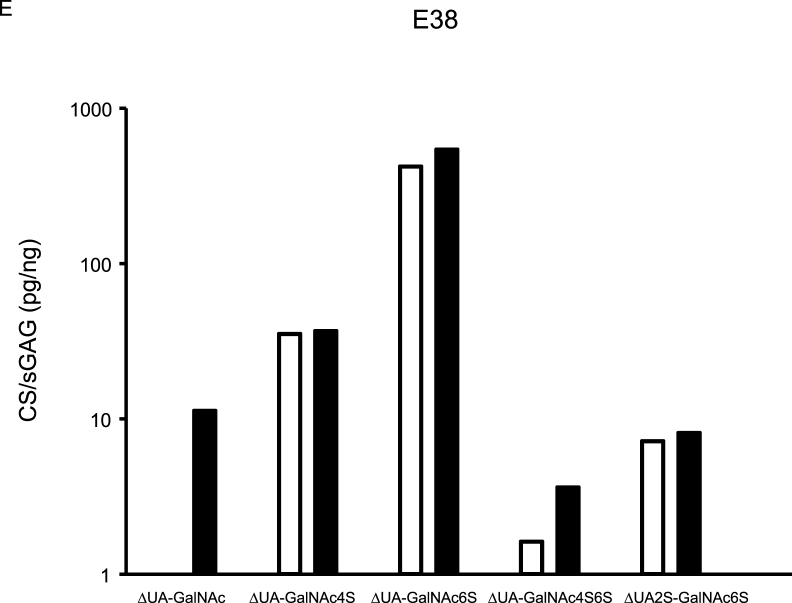
Aggrecan was purified from synovial fluid (SF) from four recent knee injury subjects (E2, E3, E33 and E38) and from the SF-control sample, and both purified aggrecan and synovial fluid samples were analyzed for chondroitin sulfate (CS) content using the HPLC-assay. Concentration is expressed as pg CS per ng sulfated glycosaminoglycan (sGAG; assessed by Alcian Blue assay). Aggrecan CS concentrations are shown as black bar graphs and synovial fluid CS concentrations as white. GalNAc = *N*-acetylgalactosamine, ΔUA ​= ​unsaturated uronic acid, 2S ​= ​sulfation on carbon atom 2 of UA, 4S6S ​= ​sulfation on carbon atoms 4 and 6 of GalNAc.

CS variants were assessed in five synovial fluids, and from aggrecan purified from the corresponding synovial fluids (Fig. 2). Of the five detected CS variants, ΔUA-GalNAc was only found in the aggrecan samples. Similar concentrations, assessed by Wilcoxon rank sum test and % recovery, of the CS variants ΔUA-GalNAc4S, ΔUA-GalNAc6S and ΔUA2S-GalNAc6S were found in synovial fluid and aggrecan samples (Fig. 2, Table S2).

### 3.6 Types of *N*-glycans found in synovial fluid

Out of the 38 subjects who donated synovial fluid samples (Table 1), synovial fluid from 17 OA subjects (mean age = 62.9 years, 35.3% women) and 12 recently knee-injured subjects (mean age = 43.6 years, 50% women) and from the SF-control were also analyzed for *N*-glycans using MS. For the patient groups analyzed by MS, there was a difference in age between OA and the “recent injury” groups, while there was no difference in sex between the groups (data not shown); although due to the low number of subjects, no adjustments were done.

In these synovial fluid samples, 61 different *N*-glycans were found (Table S3). These *N*-glycans were dived into four major classes (pauci-mannose, high-mannose, hybrid and complex) and several subclasses (core-fucose, bisecting GlcNAc, and different levels of sialylation) and were further assessed (Table 6). There was no difference in the frequency of the major classes of *N*-glycans nor in the *N*-glycan subclasses between the OA and recent injury groups; neither were there any difference in the total numbers of *N*-glycans (Table 6).

**Table 6:** Frequencies of *N*-glycans in synovial fluid. Area under the curve (AUC) per total AUC (expressed in %) was calculated for each *N*-glycan and the median for each group (OA and recent knee injury) was used for normalization (injury divided by OA).

Pauci-mannose					
		Median (min, max), %	n	Injury vs OA	
				Norm	P values
	OA	0 (0, 0.2)	17	Na	–
	Recent knee injury	0 (0, 0.2)	12	Na	0.553
High-mannose					
		Median (min, max), %	n	Injury vs OA	
				Norm	P values
	OA	2.0 (0.3, 4.2)	17	1	–
	Recent knee injury	2.2 (1.2, 4.0)	12	1.1	0.268
Hybrid					
		Median (min, max), %	n	Injury vs OA	
				Norm	P values
	OA	2.6 (0.5, 8.3)	17	1	–
	Recent knee injury	3.7 (0.6, 30.9)	12	1.42	0.211
Complex					
		Median (min, max), %	n	Injury vs OA	
				Norm	P values
	OA	94.7 (84.8, 98.0)	17	1	–
	Recent knee injury	93.7 (65.6, 97.8)	12	0.98	0.180
Core fucose					
		Median (min, max), %	n	Injury vs OA	
				Norm	P values
	OA	20.9 (9.5, 29.5)	17	1	–
	Recent knee injury	20.1 (15.2, 31.1)	12	0.96	0.556
Bisecting GlcNAc					
		Median (min, max), %	n	Injury vs OA	
				Norm	P values
	OA	15.6 (7.4, 22.8)	17	1	–
	Recent knee injury	16.9 (8.6, 21.0)	12	1.08	0.845
Total sialylation					
		Median (min, max), %	n	Injury vs OA	
				Norm	P values
	OA	77.1 (18.0, 88.7)	17	1	–
	Recent knee injury	76.1 (43.7, 90.0)	12	0.99	0.616
Mono-sialylation					
		Median (min, max), %	n	Injury vs OA	
				Norm	P values
	OA	35.8 (4.7, 43.9)	17	1	–
	Recent knee injury	37.3 (20.8, 51.1)	12	1.04	0.325
Di-sialylation					
		Median (min, max), %	n	Injury vs OA	
				Norm	P values
	OA	33.9 (11.0, 48.1)	17	1	–
	Recent knee injury	26.9 (6.9, 55.7)	12	0.79	0.347
Tri-sialylation					
		Median (min, max), %	n	Injury vs OA	
				Norm	P values
	OA	4.0 (0.9, 12.3)	17	1	–
	Recent knee injury	3.1 (0.1, 10.3)	12	0.78	0.263
Tetra-sialylation					
		Median (min, max), %	n	Injury vs OA	
				Norm	P values
	OA	0 (0, 0.4)	17	Na	–
	Recent knee injury	0.02 (0, 0.3)	12	Na	0.556
Total N-glycans					
		Median (min, max), AUC	n	Injury vs OA	
				Norm	P values
	OA	8.0 ​× ​10^5^ (1.3 ​× ​10^5^, 27.2 ​× ​10^5^)	17	1	–
	Recent knee injury	9.6 ​× ​10^5^ (3.4 ​× ​10^5^, 14.8 ​× ​10^5^)	12	1.2	0.744
Number of N-glycans					
		Median (min, max), n	n	Injury vs OA	
				Norm	P values
	OA	139 (58, 241)	17	1	–
	Recent knee injury	179 (96, 223)	12	1.29	0.376

GlcNAc = *N*-acetylglucosamine, Na ​= ​not applicable, OA ​= ​osteoarthritis.

The SF-control sample was used for the validation of the intra and inter CV of *N*-glycans (i.e., *N*-glycan release by PNGase F treatment) monitored by MS; the intra release CV was 3.3–35.9%, and the inter release CV was 0.6–44.8% (Table S4). As expected, high abundancy *N*-glycans (e.g., complex and di-sialylation) displayed low CVs (<5%), while low abundance close to detection limit (e.g., high mannose) displayed high CVs (>35%).

### 3.7 Identification of *N*-glycosylated proteins in synovial fluid

The SF-control sample was used for identification of *N*-glycosylated proteins in synovial fluid. In total, 1238 proteins were detected by MS (data not shown), and 62 of these were found to be *N*-glycosylated proteins (Table S5). Except for Collagen alpha-1 (III) chain (P02461) and Stomatin (P27105), most of the observed proteins have been previously reported as *N*-glycosylated in the UniProt database. The majority of the detected glycoproteins are commonly observed in blood preparations, i.e., carbohydrate/metal ion/heme-binding proteins, complement facto-rs, immunoglobulins, and protease inhibitors. In addition, a number of cartilage and/or synovial fluid associated proteins were observed: fibronectin (FN1, P02751), proteoglycan 4 (PRG4, Q92954), cartilage oligomeric matrix protein (COMP, P49747) and aggrecan (ACAN, P16112).

The glycosylation-sites coverage varied among the observed glycoproteins, depending on the accessibility for trypsin cleavage in the vicinity of a potential *N*-glycosylation site. All expected sites were accessible for FN1, PRG4 and COMP, whereas for aggrecan only partial *N*-glycosylation site coverage was obtained by the global glycoproteomic analysis, i.e., two in globular domain 1 (G1) and one in G2; in total nine *N*-glycosylated sites have been suggested for aggrecan ([29], Table S6).

## 4 Discussion

The technical performance of the HPLC-assay indicates that the method is well suited for quantitative analyses of CS variants and HA in synovial fluid samples. Our results suggest that the vast majority of CS disaccharides in synovial fluid (i.e., GlcA-GalNAc4S and GlcA-GalNAc6S which makes up 95% of the total CS-signal) derives from aggrecan. The CS- and HA-glycan pattern differs between OA and recently knee injured subjects and the concentrations of HA and some of the CS variants are associated with age. There were no differences between OA and recent knee injured subjects for the *N*-glycan markers assessed by MS. The low number of the glycopeptide spectrum matches observed for aggrecan suggests that aggrecan has a little contribution to the overall *N*-glycan pattern found in synovial fluid.

The technical evaluation of the HPLC assay for quantification of CS and HA disaccharides was focused on the most abundant GAG molecules found in the synovial fluid from OA and recent knee injury subjects. The intra- and-inter experiment CV levels of synovial fluid samples, CS-QC and disaccharide standards were (with few exceptions) below the for immunological assays recommended max value of 20% [30,31]. Similarly, the dilution and stability recoveries for the synovial fluid samples were (again with a few exceptions) within the acceptable range of 80–120% recommend by DeSilva and co-workers [30]. By these summarized data, we conclude that the HPLC-method is well suited for quantitative analyses of CS variants and HA in synovial fluid samples. As far as we know, this is the first time a technical performance analysis has been conducted for quantification of CS variants and HA in synovial fluid by HPLC.

There was a substantial amount of GlcA-GalNAc present in the aggrecan samples, but this CS variant was not found in the synovial fluid samples. The most likely explanation for this is that synovial fluid contains high concentrations of glucose, similar to serum concentrations [32], which eluted at the same retention time as for ΔUA-GalNAc (data not shown) concealing the CS-peak, while in the purified aggrecan samples the glucose is not present. GlcA-GalNAc4S6S was absent in one of the synovial fluid samples and was in considerably less amount in the other synovial fluid samples compared to the aggrecan samples. One explanation for this could be that the level of GlcA-GalNAc4S6S is very low (approximately 1% of the total CS-signal in SF-control sample), which makes it difficult to analyze.

The semi-quantitative method (expressed as relative units) of measuring *N*-glycans in synovial fluid did not show any differences between patient groups. The study population was small, and participants were not age-matched, which might have affected the results. Since the top 10 proteins (listed in Table S5) contributing to the *N-*glycan profile in synovial fluid are not specific for the knee joint; if these systemic proteins determine the *N*-glycan pattern in synovial fluid, this might explain the lack of differences in *N*-glycans between the joint-pathologies of OA and recent knee injury.

We found that the synovial fluid HA concentration was lower in recently knee-injured subjects compared to the levels found in age-matched OA patients, while the 6-sulfated GalNAc disaccharides (i.e., ΔUA-GalNAc6S and ΔUA2-GalNAc6S) were higher in recent knee-injured subjects than in OA subjects. Similar data using a quantitative HPLC method was also found by Shinmei and co-workers [33]. The total sGAG concentration in synovial fluid, measured by Dimethyl methylene blue (DMMB), has been shown to be higher in knee healthy subjects compared to levels in OA patients [8,9]. Our data further indicated that the total sGAG concentration in synovial fluid (measured by Alcian blue) was higher in recently knee-injured subjects than in age-matched OA subjects. While there are others that have investigated the GAG-pattern, including HA and sulfation of CS, in synovial fluid from different patient groups, it has mostly been OA, rheumatoid arthritis and healthy references that have been examined [8,9,16]. Only one paper has compared knee-injured subjects to OA patients [33], and the assessments have not been done with the less abundant CS-variants (ΔUA-GalNAc4S6S, ΔUA2S-GalNAc6S) and without a profound assay validation.

Taken together, our present study together with data from the literature, indicates that there are differences in the CS-profile and in other GAG molecules assessed in synovial fluid from different joint pathologies [2,8,9,33,34]. Others have found that the sulfation pattern of CS are related to the Kellgren-Lawrence radiographic grade of OA and that KS is related to cartilage lesions [16,18].

With increasing age, the GAG chains undergo several structural changes [2]. From birth to approximately between 20 and 30 years of age normal cartilage increases the proportion of 6-sulfated GalNAc and decreases 4-sulfated GalNAc, thereafter the proportion of 4 and 6-sulfation levels are relatively constant [11]. In the present study where we combined the OA and knee injury groups (age span 36–86 years, Table 1), the synovial fluid concentration of ΔUA-GalNAc4S did not change with increasing age, while the levels of ΔUA-GalNAc6S and total GAG decreased. In a similar study, using capillary electrophoresis to measure CS levels and using DMMB to measure total GAG in synovial fluid from adult arthritis and knee healthy reference subject, Sharif and co-workers showed that with increasing age the GalNAc4S increased, while GalNAc6S and total GAG showed no change [8]. This discrepancy between our study and that of Sharif et al. might be due to differences in the analysis methods, the age span analyzed or in patient groups used between the two studies.

Our data suggests that most of the CS in synovial fluid derive from aggrecan in the cartilage. OA is a whole-organ joint disease, and except for the articular cartilage, the menisci and the synovium could also contribute to the GAG-profile in synovial fluid. However, it has been shown that the aggrecan content, and consequently the CS content, in the menisci and synovium is far lower than in the articular cartilage [35,36]. Thus, it is likely that the vast majority of the CS in synovial fluid originates from and reflects the GAG content of the articular cartilage. Our study along with other studies also suggest that the changes in sulfation of CS may play a role in the pathogenesis of OA, for instance by affecting the proteases to degrade aggrecan [[12], [13], [14], [15],^37^]; the details on how these glycans affect aggrecanolysis are unknown. A complete assessment of the CS-profile in synovial fluid of larger cohorts with different join pathologies and disease stages is possible with our HPLC method. The use of this technique in different cohorts would thereby make it possible to search for GAG-biomarkers for prediction and/or progression of OA and other arthritides.

This study has several limitations. The synovial fluid was spun (3000×*g*) directly after aspiration and the supernatant was use for GAG-analysis; we have not checked the cell-debris pellet for GAGs. Due to the large glucose peak concealing the peak of non-sulfated CS variant (ΔUA-GalNAc), we were not able to quantify and/or measure the technical performance of this CS variant in the HPLC-assay. For this pilot study, only 38 patient-samples were used in the quantification test of the CS variants and HA and for the *N*-glycan assessments. To investigate the detailed CS/HA-profiles in different joint diseases, better-powered studies are required. The *N*-glycans, measured by MS, were due to volume limitation not measured in all samples. The *N*-glycans were semi-quantified, and for the technical validation of the *N*-glycan, intra and inter release CV assessments were done. To assure the data quality of *N*-glycans for future screening analyses of patient samples, further validation of the MS semi-quantification method could be performed.

The strength of our study is that we compared different treatments of synovial fluid for the HPLC assessment of CS and HA disaccharides, and we did a thorough evaluation of the technical performance of the HPLC-assay for assessments of four CS variants and HA.

To conclude, the HPLC-assay quantification of CS variants and HA in synovial fluid produces similar high quality and reproducible data as more commonly used immunoassays such as ELISA. This enables glycan-biomarker screening of patient samples and analysis of these molecules for experimental studies. Glycan biomarkers in clinical use could serve, among other purposes, as a screening tool to identify OA patients in early stages of the disease or to identify patients that have a certain risk of developing a severe form of the disease. They could thereby work as an aid when guiding these patients with the right preventive measures.

## Author contributions

All authors (EA, ET, SL, NK, CJ, EM, PS and AS) contributed to designing the study. EA, ET, AS, CJ, NK and EM conducted the lab-work and/or analyzed the data. All authors contributed to writing the manuscript and approved the final version

## Role of the funding source

The study was funded from the Swedish Research Council, Crafoord Foundation, the Swedish Rheumatism Association, the Faculty of Medicine at Lund University, Region Skåne Governmental funding of clinical research within the national health services (ALF), Kocks Foundation and from Alfred Österlunds Foundation. We also thank the Swedish National Infrastructure for Biological Mass Spectrometry (BioMS) for financial support of glycoproteomic studies at the Proteomics Core Facility, University of Gothenburg.

## Declaration of competing interest

All authors declare that they have no competing interests.
