# Supplementary material for: Quantification of chondroitin sulfate, hyaluronic acid and N-glycans in synovial fluid – A technical performance study
Source: Osteoarthr Cartil Open. 2023 Jun 10;5(3):100380. doi: 10.1016/j.ocarto.2023.100380 (PMC10322674; doi:10.1016/j.ocarto.2023.100380)
Supplement: Multimedia component 1 [file mmc1.docx]

**Supplemental Material to:**

**Quantification of chondroitin sulfate, hyaluronic acid and N-glycans in synovial fluid**

**Authors:**

Elin Andersson, Emil Tykesson, L. Stefan Lohmander, Niclas G Karlsson, Chunsheng Jin, Ekaterina Mirgorodskaya, Per Swärd, André Struglics

**Contents:**

Supplementary Methods………………………………………………………………....2

Supplementary Table S1………………………………………………………………....4

Supplementary Table S2………………………………………………………………....5

Supplementary Table S3…………………………………………………………………6

Supplementary Table S4………………………………………………………………....9

Supplementary Table S5………………………………………………………………....10

Supplementary Table S6…………………………………………………………………13

Supplementary Figure S1………………………………………………………………...14

Supplementary Figure S2………………………………………………………………...15

Supplementary Figure S3………………………………………………………………...16

Supplementary References……………………………………………………………….18

**SUPPLEMENTARY METHODS**

***N*-glycan assessment with mass spectrometry**

Thirty µl synovial fluid sample was diluted with 7 M urea to a final volume of 100 µl. DTT was added to 10 mM and incubated at 56°C for 45 min. The samples were alkylated by adding iodoacetamide (IAA) to 25 mM and incubating in the dark for 50 min at ambient temperature. The buffer was exchanged to 50 mM NH_4_HCO_3_, pH 8.4, using spin-filter (30 kDa cutoff, Millipore). 1 µl (10 mU) of Peptide:*N*-glycosidase F (PNGase F – CarboClip; Asparia) was added and incubated at 37°C overnight. Released N-glycans were purified, reduced and desalted as described previously [S1]. The resultant glycans were then analyzed by liquid chromatography mass spectrometry (LC-MS/MS). The samples were separated on a column of 5-µm porous graphite particles (Hypercarb, Thermo-Hypersil, Runcorn, UK; 10 cm × 250 µm packed in-house). The glycans were eluted with an acetonitrile gradient (Buffer A, 10 mM ammonium bicarbonate; Buffer B, 10 mM ammonium bicarbonate in 80% acetonitrile). The gradient (0-45% Buffer B) was eluted for 46 min, followed by a wash step with 100% Buffer B, and equilibrated with Buffer A in next 24 min. The samples were analyzed in both negative-ion mode on a LTQ linear ion trap MS (Thermo Electron, San José, CA), Data acquisition and processing were conducted with Xcalibur software (Version 2.0.7).

Glycans were identified from their MS/MS spectra by manual annotation. The biosynthesis of *N*-glycans was assumed to follow the classical pathways. Chain elongation was expected to be mediated by the addition of *N*-acetyllactosamine units. Diagnostic fragmentation ions for *N*-glycans were investigated as described [S2]. The frequency of the *N*-glycans in the synovial fluid samples were calculated as the area under the curve (AUC) of each *N*-glycan structure divided by the total AUC expressed as a percentage. The peak area was processed by Progenesis QI (Nonlinear Dynamics, Newcastle, UK).

**Global analysis of *N*-glycosylated proteins in synovial fluid**

Synovial fluid control sample (1 mg) in 50 mM triethylammonium bicarbonate pH 8 (TEAB), 2% sodium deoxycholate (SDC), was reduced with DTT (5 mM, 56°C, 30 min) and alkylated with IAA (10 mM, 30 min, room temperature (RT)). The alkylation reaction was quenched by incubation with DTT (10 mM, 15 min, RT). The SDC concentration was reduced to 0.5 % with 50 mM TEAB and sample was digested with Pierce MS grade trypsin, first with 10 µg for 4 h followed by an extra addition of 10 µg trypsin and an overnight incubation, both at 37°C. Digestion was stopped and SDC was removed by acidification and subsequent centrifugation. The supernatant was further purified using Pierce peptide desalting spin columns (Thermo Fisher Scientific, # 89852) followed by glycopeptide enrichment using in-house prepared hydrophilic interaction liquid chromatography (HILIC) spin columns (20 mg ZIC-HILIC resins per column, SeQuant # 2942). Both HILIC flow-through and eluate were collected and used for further proteomic and glycoproteomic analysis, respectively. HILIC flow-through fraction was further fractionated by basic reversed-phase chromatography (XBridge BEH C18, 3.5 μm, 3.0x150 mm, Waters Corporation) using a Dionex Ultimate 3000 UPLC system (Thermo Fischer Scientific) and a gradient from 3% to 90% acetonitrile in 10 mM ammonium formate at pH 10.00 over 25 min. The 40 primary fractions were concatenated into 16 fractions. For nanoLC-MS, the HILIC eluate and the HILIC flow-through fractions were reconstituted in 2% acetonitrile (ACN), 0.2% formic acid (FA).

NanoLC-MS was performed on QExactive HF mass spectrometer interfaced with Easy-nLC1200 liquid chromatography system (Thermo Fisher Scientific). Peptides were trapped on an Acclaim Pepmap 100 C18 trap column (100 μm x 2 cm, particle size 5 μm, Thermo Fischer Scientific) and separated on an in-house packed analytical column (75 μm x 300 mm, particle size 3 μm, Reprosil-Pur C18, Dr. Maisch).

HILIC flow-through fractions were analyzed using a 75 min gradient from 5% to 35% ACN in 0.1% FA. MS scans were acquired in *m/z* 400-1600 range at a resolution of 60K. MS/MS analysis was performed in a data-dependent mode at a resolution of 30K. The 10 most intense ions, charge states 2 to 4, were selected for fragmentation using higher-energy C-trap dissociation (HCD) at collision energy settings of 28. The isolation window was set to *m/z* 1.2 and dynamic exclusion to 10 ppm for 20 s.

The HILIC eluate preparation, enriched in glycopeptides, was analyses using a 110 min gradient from 4% to 40% ACN in 0.1% FA. Multiple injections were acquired, all with precursors detection in *m/z* 600-2000 range at a resolution of 120K, but with different fragmentation energy settings. MS/MS analysis was always performed in a data-dependent mode. The 10 most intense ions with charge states 2 to 5 were selected for fragmentation using HCD at collision energy settings of either 24, 28, 30 or 38. The isolation window was set to *m/z* 3 and dynamic exclusion set to 10 ppm for 20 s.

The data were analyzed using Proteome Discoverer version 2.4 (Thermo Fisher Scientific). First, the data from HILIC flow-through fractions were matched against Swiss-Prot *Homo sapiens* database, using the Mascot search engine v. 2.5.1 (Matrix Science, London, UK). The precursor mass tolerance was set to 5 ppm and fragment mass tolerance to 30 mmu. Tryptic peptides were accepted with one missed cleavage, fixed modification for cysteine alkylation and variable modifications for methionine oxidation. Percolator was used for peptide spectrum matches (PSM) validation with the strict false discovery rate (FDR) threshold of 1%. This resulted in identification of 1238 proteins at 1% FDR. Those were used as a new database for the glycoproteomic search of the data acquired from HILIC enriched preparation. The data from HILIC enriched preparation were analyzed with Byonic (Protein Metrics) as search engines. The *N*-glycan composition database contained in total 46 compositions, of which 42 compositions were based on the glycomic analysis and 4 compositions were virtual ones suggested by the GlyConnect Compozitor version 1.0.0 (www.expasy.org). Precursor mass tolerance was set to 10 ppm and fragment mass tolerance to 30 ppm. Tryptic peptides with up to 2 missed cleavages were accepted together with fixed cysteine alkylation, variable modification of methionine oxidation and variable *N*-glycosylation carrying glycans defined in the *N*-glycan database. The suggested glycosylated peptide identifications were evaluated based on the number of glycoforms per each site, number of PSM per glycoform (calculated as sum of all PSMs in four independent injection) and the retention time window for the observed glycoforms with the same peptide core. The glycoforms identified based on single PSM were automatically rejected. All glycoforms, that were assigned to sites with more than one identified glycoform per site and identified with 3 or more PSMs, were automatically accepted. The remaining glycopeptide identifications were manually validated based on the consistency with expected fragmentation pattern and retention times.

Table S1. Injection of lower volume of synovial fluid control sample and test of boiling treatment. Five times lower synovial fluid sample volume (0.028 µl; A1-low and C1-low) compared to normal injection volume (0.14 µl; A1) was injected into the HPLC. Duplicates per treatment was used, and the data is expressed as relative units of mean values of A1 or A1-low. CV for duplicates are shown as text below.

|  | **∆UA-GalNAc4S** | | **∆UA-GalNAc6S** | | **∆UA-GalNAc4S6S** | |
| --- | --- | --- | --- | --- | --- | --- |
| A1 | *1* |  | *1* |  | *1* |  |
| A1-low | 1.48 | *1* | 1.23 | *1* | < LLOD | *1* |
| C1-low | 1.24 | 0.84 | 1.11 | 0.90 | < LLOD | Na |

Treatments, for an overview see Figure S1, (CV-range for the three chondroitin sulfates, mean values from duplicates): A1, chondroitinase (2.2-56.7%); A1-low, chondroitinase (7.5-19.2%); C1-low, chondroitinase + boiling (2.9%, 6.0%). ∆UA = unsaturated uronic acid, GalNAc = *N*-acetylgalactosamine, < LLOD = below lower limit of detection (no peak), CV = coefficient of variation, HPLC = high performance liquid chromatography, Na = not applicable, 2S = sulfation on carbon atom 2 of UA, 4S6S = sulfation on carbon atoms 4 and 6 of GalNAc.

Table S2. Chondroitin sulfate (CS) content in synovial fluid (SF) and on aggrecan purified from same patients and SF-control were quantified by the HPLC-assay. The mean concentration from duplicate injections, expressed as pg CS per ng sulfated glycosaminoglycan (sGAG), are shown. The p-values (Wilcoxon signed-rank test) for group comparisons (SF vs aggrecan) for each CS-marker are presented.

| **∆UA-GalNAc** |  |  |  |  |
| --- | --- | --- | --- | --- |
| (p = na) |  |  |  |  |
|  | Samples | SF | Aggrecan | SF/aggrecan |
|  |  | (pg CS/ng GAG) | (pg CS/ng GAG) | (%) |
|  | Control | - | 39,81 | Na |
|  | E2 | - | 24,05 | Na |
|  | E3 | - | 21,88 | Na |
|  | E33 | - | 62,11 | Na |
|  | E38 | - | 11,32 | Na |
| **∆UA-GalNAc4S** |  |  |  |  |
| (p = 0.500) |  |  |  |  |
|  | Samples | SF | Aggrecan | SF/aggrecan |
|  |  | (pg CS/ng GAG) | (pg CS/ng GAG) | (%) |
|  | Control | 68,23 | 49,69 | 137,30 |
|  | E2 | 50,44 | 62,49 | 80,71 |
|  | E3 | 26,00 | 32,37 | 80,34 |
|  | E33 | 83,90 | 100,53 | 83,46 |
|  | E38 | 35,81 | 36,89 | 97,08 |
| **∆UA-GalNAc6S** |  |  |  |  |
| (p = 0.080) |  |  |  |  |
|  | Samples | SF | Aggrecan | SF/aggrecan |
|  |  | (pg CS/ng GAG) | (pg CS/ng GAG) | (%) |
|  | Control | 681,39 | 612,95 | 111,17 |
|  | E2 | 442,99 | 671,61 | 65,96 |
|  | E3 | 473,62 | 682,63 | 69,38 |
|  | E33 | 588,41 | 853,15 | 68,97 |
|  | E38 | 429,25 | 545,15 | 78,74 |
| **∆UA-GalNAc4S6S** |  |  |  |  |
| (p = 0.068) |  |  |  |  |
|  | Samples | SF | Aggrecan | SF/aggrecan |
|  |  | (pg CS/ng GAG) | (pg CS/ng GAG) | (%) |
|  | Control | 2,24 | 5,74 | 39,06 |
|  | E2 | 2,24 | 10,15 | 22,03 |
|  | E3 | - | 5,77 | Na |
|  | E33 | 3,06 | 12,69 | 24,11 |
|  | E38 | 1,65 | 3,64 | 45,29 |
| **∆UA2S-GalNAc6S** |  |  |  |  |
| (p = 0.138) |  |  |  |  |
|  | Samples | SF | Aggrecan | SF/aggrecan |
|  |  | (pg CS/ng GAG) | (pg CS/ng GAG) | (%) |
|  | Control | 11,73 | 6,79 | 172,7 |
|  | E2 | 10,33 | 7,89 | 130,9 |
|  | E3 | 5,95 | 5,59 | 106,5 |
|  | E33 | 10,35 | 8,80 | 117,6 |
|  | E38 | 7,30 | 8,18 | 89,3 |

∆UA = unsaturated uronic acid, GalNAc = *N*-acetylgalactosamine, 2S = sulfation on carbon atom 2 of UA, 4S6S = sulfation on carbon atoms 4 and 6 of GalNAc, - = no data/no signal obtained, Na = not applicable.

Table S3. Different types of *N*-glycans found in synovial fluid OA (n = 17) and recent knee injury (n = 12) samples.

| **#** | **Mass** | **Composition** | **Putative structure** |
| --- | --- | --- | --- |
| 1 | 425 | HexNAc2 | GlcNAcβ1-4GlcNAcol |
| 2 | 1114 | Hex3HexNAc3 | GlcNAcβ1-2Manα1-3(Manα1-6)Manβ1-4GlcNAcβ1-4GlcNAcol |
| 3 | 1235 | Hex5HexNAc2 | Manα1-3[Manα1-3(Manα1-6)Manα1-6]Manβ1-4GlcNAcβ1-4GlcNAcol |
| 4 | 1317 | Hex3HexNAc4 | GlcNAcβ1-2Manα1-3(GlcNAcβ1-2Manα1-6)Manβ1-4GlcNAcβ1-4GlcNAcol |
| 5 | 1397 | Hex6HexNAc2 | Manα1-2Manα1-3[Manα1-3(Manα1-6)Manα1-6]Manβ1-4GlcNAcβ1-4GlcNAcol |
| 6 | 1463-1 | Hex3HexNAc4deHex1 | GlcNAcβ1-2Manα1-3(Manα1-6)(GlcNAcβ1-4)Manβ1-4GlcNAcβ1-4(Fucα1-6)GlcNAcol |
| 7 | 1463-2 | Hex3HexNAc4deHex1 | GlcNAcβ1-2Manα1-3(GlcNAcβ1-2Manα1-6)Manβ1-4GlcNAcβ1-4(Fucα1-6)GlcNAcol |
| 8 | 1479 | Hex4HexNAc4 | GlcNAcβ1-2Manα1-3(Galβ1-4GlcNAcβ1-2Manα1-6)Manβ1-4GlcNAcβ1-4GlcNAcol |
| 9 | 1520 | Hex3HexNAc5 | GlcNAcβ1-2Manα1-3(GlcNAcβ1-4)(GlcNAcβ1-2Manα1-6)Manβ1-4GlcNAcβ1-4GlcNAcol |
| 10 | 1559 | Hex7HexNAc2 | Manα1-2Manα1-3[Manα1-2Manα1-3(Manα1-6)Manα1-6]Manβ1-4GlcNAcβ1-4GlcNAcol |
| 11 | 1567 | NeuAc1Hex4HexNAc5 | NeuAcα2-6GlcNAcβ1-2Manα1-3(Manα1-6)Manβ1-4GlcNAcβ1-4GlcNAcol |
| 12 | 1600 | Hex6HexNAc3 | Galβ1-4GlcNAcβ1-2Manα1-3[Manα1-3(Manα1-6)Man α1-6]Manβ1-4GlcNAcβ1-4GlcNAcol |
| 13 | 1625-1 | Hex4HexNAc4deHex1 | Galβ1-4GlcNAcβ1-2Manα1-3(GlcNAcβ1-2Manα1-6)Manβ1-4GlcNAcβ1-4(Fucα1-6)GlcNAcol |
| 14 | 1625-2 | Hex4HexNAc4deHex1 | GlcNAcβ1-2Manα1-3(Galβ1-4GlcNAcβ1-2Manα1-6)Manβ1-4GlcNAcβ1-4(Fucα1-6)GlcNAcol |
| 15 | 1641 | Hex5HexNAc4 | Galβ1-4GlcNAcβ1-2Manα1-3(Galβ1-4GlcNAcβ1-2Manα1-6)Manβ1-4GlcNAcβ1-4GlcNAcol |
| 16 | 1666 | Hex3HexNAc5deHex1 | GlcNAcβ1-2Manα1-3(GlcNAcβ1-4)(GlcNAcβ1-2Manα1-6)Manβ1-4GlcNAcβ1-4(Fucα1-6)GlcNAcol |
| 17 | 1682 | Hex4HexNAc5 | GlcNAcβ1-2Manα1-3(GlcNAcβ1-4)(Galβ1-4GlcNAcβ1-2Manα1-6)Manβ1-4GlcNAcβ1-4GlcNAcol |
| 18 | 1721 | Hex8HexNAc2 | Manα1-2Manα1-2Manα1-3[Manα1-2Manα1-3(Manα1-6)Manα1-6]Manβ1-4GlcNAcβ1-4GlcNAcol |
| 19 | 1729-1 | NeuAc1Hex5HexNAc3 | NeuAcα2-3Galβ1-4GlcNAcβ1-2Manα1-3(Manα1-6Manα1-6)Manβ1-4GlcNAcβ1-4GlcNAcol |
| 20 | 1729-2 | NeuAc1Hex5HexNAc3 | NeuAcα2-3Galβ1-4GlcNAcβ1-2Manα1-3(Manα1-3Manα1-6)Manβ1-4GlcNAcβ1-4GlcNAcol |
| 21 | 1770-1 | NeuAc1Hex4HexNAc4 | GlcNAcβ1-2Manα1-3(NeuAcα2-6Galβ1-4GlcNAcβ1-2Manα1-6)Manβ1-4GlcNAcβ1-4GlcNAcol |
| 22 | 1770-2 | NeuAc1Hex4HexNAc4 | GlcNAcβ1-2Manα1-3(NeuAcα2-3Galβ1-4GlcNAcβ1-2Manα1-6)Manβ1-4GlcNAcβ1-4GlcNAcol |
| 23 | 1770-3 | NeuAc1Hex4HexNAc4 | NeuAcα2-3Galβ1-4GlcNAcβ1-2Manα1-3(GlcNAcβ1-2Manα1-6)Manβ1-4GlcNAcβ1-4GlcNAcol |
| 24 | 1787 | Hex5HexNAc4dHex1 | Galβ1-4GlcNAcβ1-2Manα1-3(Galβ1-4GlcNAcβ1-2Manα1-6)Manβ1-4GlcNAcβ1-4(Fucα1-6)GlcNAcol |
| 25 | 1828 | Hex4HexNAc5deHex1 | GlcNAcβ1-2Manα1-3(GlcNAcβ1-4)(Galβ1-4GlcNAcβ1-2Manα1-6)Manβ1-4GlcNAcβ1-4(Fucα1-6)GlcNAcol |
| 26 | 1844 | Hex5HexNAc5 | Galβ1-4GlcNAcβ1-2Manα1-3(GlcNAcβ1-4)(Galβ1-4GlcNAcβ1-2Manα1-6)Manβ1-4GlcNAcβ1-4GlcNAcol |
| 27 | 1883 | Hex9HexNAc2 | Manα1-2Manα1-2Manα1-3[Manα1-2Manα1-3(Manα1-2Manα1-6)Manα1-6]Manβ1-4GlcNAcβ1-4GlcNAcol |
| 28 | 1891 | NeuAc1Hex6HexNAc3 | NeuAcα2-6Galβ1-4GlcNAcβ1-2Manα1-3[Manα1-3(Manα1-6)Manα1-6]Manβ1-4GlcNAcβ1-4GlcNAcol |
| 29 | 1916 | NeuAc1Hex4HexNAc4deHex1 | GlcNAcβ1-2Manα1-3(NeuAcα2-6Galβ1-4GlcNAcβ1-2Manα1-6)Manβ1-4GlcNAcβ1-4(Fucα1-6)GlcNAcol |
| 30 | 1932 | NeuAc1Hex5HexNAc4 | NeuAcα2-3Galβ1-4GlcNAcβ1-2Manα1-3(Galβ1-4GlcNAcβ1-2Manα1-6)Manβ1-4GlcNAcβ1-4GlcNAcol |
| 31 | 1932 | NeuAc1Hex5HexNAc4 | Galβ1-4GlcNAcβ1-2Manα1-3(NeuAcα2-3Galβ1-4GlcNAcβ1-2Manα1-6)Manβ1-4GlcNAcβ1-4GlcNAcol |
| 32 | 1973 | NeuAc1Hex4HexNAc5 | NeuAc + GlcNAcβ1-2Manα1-3(GlcNAcβ1-4)(Galβ1-4GlcNAcβ1-2Manα1-6)Manβ1-4GlcNAcβ1-4GlcNAcol |
| 33 | 1990 | Hex5HexNAc5deHex1 | Galβ1-4GlcNAcβ1-2Manα1-3(GlcNAcβ1-4)(Galβ1-4GlcNAcβ1-2Manα1-6)Manβ1-4GlcNAcβ1-4(Fucα1-6)GlcNAcol |
| 34 | 2006-1 | Hex6HexNAc5 | Galβ1-4GlcNAcβ1-2Manα1-3[Galβ1-4GlcNAcβ1-2(Galβ1-4GlcNAcβ1-6)Manα1-6]Manβ1-4GlcNAcβ1-4GlcNAcol |
| 35 | 2006-2 | Hex6HexNAc5 | Galβ1-4GlcNAcβ1-2(Galβ1-4GlcNAcβ1-4)Manα1-3(Galβ1-4GlcNAcβ1-2Manα1-6)Manβ1-4GlcNAcβ1-4GlcNAcol |
| 36 | 2078 | NeuAc1Hex5HexNAc4dHex1 | NeuAcα2-6Galβ1-4GlcNAcβ1-2Manα1-3(Galβ1-4GlcNAcβ1-2Manα1-6)Manβ1-4GlcNAcβ1-4(Fucα1-6)GlcNAcol |
| 37 | 2135 | NeuAc1Hex5HexNAc5 | NeuAcα2-6Galβ1-4GlcNAcβ1-2Manα1-3(GlcNAcβ1-4)(Galβ1-4GlcNAcβ1-2Manα1-6)Manβ1-4GlcNAcβ1-4GlcNAcol |
| 38 | 2223-1 | NeuAc2Hex5HexNAc4 | NeuAcα2-6Galβ1-4GlcNAcβ1-2Manα1-3(NeuAcα2-6Galβ1-4GlcNAcβ1-2Manα1-6)Manβ1-4GlcNAcβ1-4GlcNAcol |
| 39 | 2223-2 | NeuAc2Hex5HexNAc4 | NeuAcα2-6Galβ1-4GlcNAcβ1-2Manα1-3(NeuAcα2-3Galβ1-4GlcNAcβ1-2Manα1-6)Manβ1-4GlcNAcβ1-4GlcNAcol |
| 40 | 2281-1 | NeuAc1Hex5HexNAc5deHex1 | NeuAcα2-6Galβ1-4GlcNAcβ1-2Manα1-3(GlcNAcβ1-4)(Galβ1-4GlcNAcβ1-2Manα1-6)Manβ1-4GlcNAcβ1-4(Fucα1-6)GlcNAcol |
| 41 | 2281-2 | NeuAc1Hex5HexNAc5deHex1 | NeuAcα2-6Galβ1-4GlcNAcβ1-2Manα1-3(GlcNAcβ1-4)(Galβ1-4GlcNAcβ1-2Manα1-6)Manβ1-4GlcNAcβ1-4(Fucα1-6)GlcNAcol |
| 42 | 2297-1 | NeuAc1Hex6HexNAc5 | NeuAc+Galβ1-4GlcNAcβ1-2(Galβ1-4GlcNAcβ1-6)Manα1-3(Galβ1-4GlcNAcβ1-2Manα1-6)Manβ1-4GlcNAcβ1-4GlcNAcol |
| 43 | 2297-2 | NeuAc1Hex6HexNAc5 | NeuAc+Galβ1-4GlcNAcβ1-2(Galβ1-4GlcNAcβ1-6)Manα1-3(Galβ1-4GlcNAcβ1-2Manα1-6)Manβ1-4GlcNAcβ1-4GlcNAcol |
| 44 | 2297-3 | NeuAc1Hex6HexNAc5 | NeuAc+Galβ1-4GlcNAcβ1-2(Galβ1-4GlcNAcβ1-6)Manα1-3(Galβ1-4GlcNAcβ1-2Manα1-6)Manβ1-4GlcNAcβ1-4GlcNAcol |
| 45 | 2369 | NeuAc2Hex5HexNAc4dHex1 | NeuAcα2-6Galβ1-4GlcNAcβ1-2Manα1-3(NeuAcα2-3Galβ1-4GlcNAcβ1-2Manα1-6)Manβ1-4GlcNAcβ1-4(Fucα1-6)GlcNAcol |
| 46 | 2426 | NeuAc2Hex5HexNAc5 | NeuAcα2-6Galβ1-4GlcNAcβ1-2Manα1-3(GlcNAcβ1-4)(NeuAcα2-6Galβ1-4GlcNAcβ1-2Manα1-6)Manβ1-4GlcNAcβ1-4GlcNAcol |
| 47 | 2572 | NeuAc2Hex5HexNAc5deHex1 | NeuAcα2-6Galβ1-4GlcNAcβ1-2Manα1-3(GlcNAcβ1-4)(NeuAcα2-6Galβ1-4GlcNAcβ1-2Manα1-6)Manβ1-4GlcNAcβ1-4(Fucα1-6)GlcNAcol |
| 48 | 2588-1 | NeuAc2Hex6HexNAc5 | 2NeuAc+Galβ1-4GlcNAcβ1-2(Galβ1-4GlcNAcβ1-6)Manα1-3(Galβ1-4GlcNAcβ1-2Manα1-6)Manβ1-4GlcNAcβ1-4GlcNAcol |
| 49 | 2588-2 | NeuAc2Hex6HexNAc5 | 2NeuAc+Galβ1-4GlcNAcβ1-2(Galβ1-4GlcNAcβ1-6)Manα1-3(Galβ1-4GlcNAcβ1-2Manα1-6)Manβ1-4GlcNAcβ1-4GlcNAcol |
| 50 | 2734 | NeuAc2Hex6HexNAc5dHex1 | 2xNeuAcα2-3+ Galβ1-4GlcNAcβ1-2(Galβ1-4GlcNAcβ1-4)Manα1-3(Galβ1-4GlcNAcβ1-2Manα1-6)Manβ1-4GlcNAcβ1-4(Fucα1-6)GlcNAcol |
| 51 | 2880-1 | NeuAc3Hex6HexNAc5 | NeuAcα2-6Galβ1-4GlcNAcβ1-2(NeuAcα2-6Galβ1-4GlcNAcβ1-6)Manα1-3(NeuAcα2-6Galβ1-4GlcNAcβ1-2Manα1-6)Manβ1-4GlcNAcβ1-4GlcNAcol |
| 52 | 2880-2 | NeuAc3Hex6HexNAc5 | NeuAcα2-6Galβ1-4GlcNAcβ1-2(NeuAcα2-6Galβ1-4GlcNAcβ1-6)Manα1-3(NeuAcα2-6Galβ1-4GlcNAcβ1-2Manα1-6)Manβ1-4GlcNAcβ1-4GlcNAcol |
| 53 | 2880-3 | NeuAc3Hex6HexNAc5 | NeuAcα2-6Galβ1-4GlcNAcβ1-2(NeuAcα2-6Galβ1-4GlcNAcβ1-6)Manα1-3(NeuAcα2-6Galβ1-4GlcNAcβ1-2Manα1-6)Manβ1-4GlcNAcβ1-4GlcNAcol |
| 54 | 2954 | NeuAc2Hex7HexNAc6 | ND |
| 55 | 3026-1 | NeuAc3Hex6HexNAc5dHex1 | 2NeuAcα2-3 + NeuAcα2-6 + Galβ1-4GlcNAcβ1-2(Galβ1-4GlcNAcβ1-4)Manα1-3(Galβ1-4GlcNAcβ1-2Manα1-6)Manβ1-4GlcNAcβ1-4(Fucα1-6)GlcNAcol |
| 56 | 3026-2 | NeuAc3Hex6HexNAc5dHex1 | NeuAcα2-3Galβ1-4GlcNAcβ1-2(NeuAcα2-3Galβ1-4GlcNAcβ1-4)Manα1-3(NeuAcα2-3Galβ1-4GlcNAcβ1-2Manα1-6)Manβ1-4GlcNAcβ1-4(Fucα1-6)GlcNAcol |
| 57 | 3026-3 | NeuAc3Hex6HexNAc5dHex1 | NeuAcα2-3Galβ1-4GlcNAcβ1-2(NeuAcα2-3Galβ1-4GlcNAcβ1-4)Manα1-3(NeuAcα2-3Galβ1-4GlcNAcβ1-2Manα1-6)Manβ1-4GlcNAcβ1-4(Fucα1-6)GlcNAcol |
| 58 | 3254-1 | NeuAc3Hex7HexNAc6 | ND |
| 59 | 3254-2 | NeuAc3Hex7HexNAc6 | ND |
| 60 | 3536-1 | NeuAc4HexNAc7HexNAc6 | ND |
| 61 | 3536-2 | NeuAc4HexNAc7HexNAc6 | ND |

Fuc = Fucose, Gal = Galactose, GlcNAc = *N*-acetylglucosamine, GlcNAcol = *N*-acetylglucosaminitol, Hex = Hexose, Man = Mannose, NAc = *N*-acetyl, ND = not possible to determine structure, NeuAc = *N*-acetylneuraminic acid, OA = osteoarthritis.

Table S4. Coefficient of variation (CV) of *N*-glycan release and quantification by mass spectrometry. For intra release, the synovial fluid control sample was digested with Peptide:*N*-glycosidase F (PNGase F) and was injected consecutively three times into mass spectrometer. For inter release, three separate PNGase F digestions of the synovial fluid control sample were done, and these were then injected consecutively into the mass spectrometer. The data is expressed as area under the curve (AUC) per total AUC in %.

|  | **Intra release** |  | **Inter release** |  |
| --- | --- | --- | --- | --- |
|  | AUC/total AUC, % | CV, % | AUC/total AUC, % | CV, % |
| High mannose | 1.3 | 35.9 | 0.9 | 44.8 |
| Complex | 96.3 | 4.8 | 99.1 | 0.4 |
| Core fucose | 16.7 | 8.4 | 18.9 | 3.2 |
| Bisecting GlcNAc | 4.1 | 21.6 | 4.2 | 18.0 |
| Total sialylation | 94.1 | 4.9 | 96.4 | 0.8 |
| Mono-sialylation | 12.7 | 3.3 | 13.2 | 2.7 |
| Di-sialylation | 68.0 | 7.0 | 68.7 | 0.6 |
| Tri-sialylation | 13.4 | 11.2 | 14.4 | 4.6 |

Table S5. *N*-glycosylated proteins identified by mass spectrometry in the synovial fluid control sample. The proteins are listed in order of total number of glycopeptide spectrum matches (glycoPSM) for a given protein, in the hydrophilic interaction liquid chromatography enriched preparation. Glycosite is defined by a unique amino acid sequence, and Glycoform is defined by either a unique amino acid sequence, a unique glycan composition, or both. glycoPSMs = PSMs associated exclusively with glycopeptides, evident by the presence of oxonium ions. Cartilage and/or synovial fluid associated proteins are marked with bold font.

| **Accession number** | **Protein (gene name)** | **Number of glycosites** | **Number of glycoforms (sum of all sites)** | **Number of glycoPSMs (sum of all glycoforms)** |
| --- | --- | --- | --- | --- |
| P02763 | Alpha-1-acid glycoprotein 1 (ORM1) | 4 | 36 | 631 |
| P00738 | Haptoglobin (HP) | 3 | 36 | 541 |
| P01871 | Immunoglobulin heavy constant mu (IGHM) | 5 | 65 | 509 |
| P00450 | Ceruloplasmin (CP) | 3 | 26 | 476 |
| P02787 | Serotransferrin (TF) | 2 | 16 | 412 |
| P02790 | Hemopexin (HPX) | 2 | 16 | 346 |
| P01859 | Immunoglobulin heavy constant gamma 2 (IGHG2) | 3 | 24 | 340 |
| P0DOX5 | Immunoglobulin gamma-1 heavy chain (?) | 1 | 16 | 307 |
| P02765 | Alpha-2-HS-glycoprotein (AHSG) | 3 | 18 | 282 |
| P04196 | Histidine-rich glycoprotein (GN) | 2 | 13 | 280 |
| P00734 | Prothrombin (F2) | 5 | 17 | 246 |
| P04004 | Vitronectin (VTN) | 3 | 19 | 221 |
| P01009 | Alpha-1-antitrypsin (SERPINA1) | 1 | 9 | 220 |
| P01042 | Kininogen-1 (KNG1) | 5 | 25 | 187 |
| P01024 | Complement C3 (C3) | 2 | 12 | 184 |
| P05546 | Heparin cofactor 2 (SERPIND1) | 3 | 15 | 179 |
| **P02751** | **Fibronectin (FN1)** | **5** | **27** | **178** |
| P01011 | Alpha-1-antichymotrypsin (SERPINA3) | 2 | 12 | 151 |
| P04114 | Apolipoprotein B-100 (APOB) | 5 | 23 | 150 |
| P08603 | Complement factor H (CFH) | 4 | 16 | 143 |
| P01861 | Immunoglobulin heavy constant gamma 4 (IGHG4) | 1 | 10 | 131 |
| P25311 | Zinc-alpha-2-glycoprotein (AZGP1) | 1 | 6 | 123 |
| P02749 | Beta-2-glycoprotein 1 (APOH) | 2 | 8 | 119 |
| P01876 | Immunoglobulin heavy constant alpha 1 (IGHA1) | 1 | 11 | 115 |
| P02675 | Fibrinogen beta chain (FGB) | 1 | 5 | 113 |
| **Q92954** | **Proteoglycan 4 (PRG4)** | **2** | **13** | **107** |
| P01591 | Immunoglobulin J chain (JCHAIN) | 2 | 13 | 103 |
| P27169 | Serum paraoxonase/arylesterase 1 (PON1) | 2 | 12 | 98 |
| P43652 | Afamin (AFM) | 3 | 9 | 90 |
| P0C0L5 | Complement C4-B (C4B) | 2 | 5 | 87 |
| **P49747** | **Cartilage oligomeric matrix protein (COMP)** | **2** | **9** | **84** |
| P01008 | Antithrombin-III (SERPINC1) | 2 | 6 | 84 |
| P10909 | Clusterin (CLU) | 1 | 8 | 81 |
| P05156 | Complement factor I (CFI) | 4 | 9 | 73 |
| P05090 | Apolipoprotein D (APOD) | 1 | 8 | 68 |
| P01019 | Angiotensinogen (AGT) | 2 | 9 | 66 |
| P00748 | Coagulation factor XII (F12) | 3 | 8 | 60 |
| **P16112** | **Aggrecan core protein (ACAN)** | **3** | **6** | **56** |
| P01031 | Complement C5 (C5) | 1 | 9 | 51 |
| P05155 | Plasma protease C1 inhibitor (SERPING1) | 2 | 12 | 48 |
| O75882 | Attractin (ATRN) | 2 | 8 | 37 |
| P19652 | Alpha-1-acid glycoprotein 2 (ORM2) | 1 | 9 | 26 |
| P51884 | Lumican (LUM) | 1 | 5 | 25 |
| P13671 | Complement component C6 (C6) | 2 | 3 | 20 |
| P03952 | Plasma kallikrein (KLKB1) | 1 | 3 | 18 |
| O95445 | Apolipoprotein M (APOM) | 1 | 3 | 17 |
| P19827 | Inter-alpha-trypsin inhibitor heavy chain H1 (ITIH1) | 1 | 2 | 16 |
| P02461 | Collagen alpha-1(III) chain (COL3A1) | 1 | 3 | 15 |
| P27105 | Stomatin (STOM) | 1 | 2 | 13 |
| P05543 | Thyroxine-binding globulin (SERPINA7) | 1 | 2 | 12 |
| P02679 | Fibrinogen gamma chain (FGG) | 1 | 2 | 12 |
| P20851 | C4b-binding protein beta chain (C4BPB) | 1 | 3 | 9 |
| Q03591 | Complement factor H-related protein 1 (CFHR1) | 1 | 2 | 9 |
| Q9UGM5 | Fetuin-B (FETUB) | 1 | 2 | 6 |
| P07738 | Bisphosphoglycerate mutase (BPGM) | 1 | 2 | 5 |
| P05154 | Plasma serine protease inhibitor (SERPINA5) | 1 | 1 | 5 |
| P55058 | Phospholipid transfer protein (PLTP) | 1 | 2 | 4 |
| P08123 | Collagen alpha-2(I) chain (COL1A2) | 1 | 2 | 4 |
| P05164 | Myeloperoxidase (MPO) | 1 | 2 | 3 |
| P26927 | Hepatocyte growth factor-like protein (MST1) | 1 | 1 | 3 |
| P00751 | Complement factor B (CFB) | 1 | 2 | 2 |
| P27918 | Properdin (CFP) | 1 | 1 | 1 |

Table S6. *N*-glycosylation sites on human aggrecan.

| ^A^**Position** | ^B^***N*-glycan composition** | **Aggrecan domain** | **Comments** |
| --- | --- | --- | --- |
| ^126^NDS | HexNAc(2)Hex(5) | G1-A | This study |
|  | HexNAc(2)Hex(6) | G1-A | This study |
| ^239^NET | Nd | G1-B | Potential site based on sequence |
| ^333^NQT | HexNAc(3)Hex(5)NeuAc(1) | G1-B’ | This study |
|  | HexNAc(4)Hex(5)dHex(1) | G1-B’ | This study |
|  | HexNAc(4)Hex(5)dHex(1)NeuAc(1) | G1-B’ | This study |
| ^387^NIT | Nd | IGD | Potential site based on sequence |
| ^434^NET | Nd | IGD | Potential site based on sequence |
| ^602^NAT | Nd | G2-B’ | Potential site based on sequence |
| ^658^NQT | HexNAc(4)Hex(5)dHex(1)NeuAc(1) | G2-B’ | This study, reference [S3] |
| ^738^NQT | Nd | KS | Potential site based on sequence |
| ^2013^NQT | Nd | CS2 | Potential site based on sequence |

(A) The attachment site of *N*-glycans to asparagine (N). The amino acid numbers are based on full-length sequence of human aggrecan (NCBI accession number P16112.3). (B) For the position ^126^N and ^333^N, two respectively three different *N*-glycans were observed. CS2 = chondroitin sulfate region 2, G1-A = loop A of globular domain 1, G1-B’ = loop B’ of globular domain 1, Hex = Hexose, dHex = Deoxy-hexose, IGD = inter globular domain, KS = keratan sulfate region, NAc = *N*-acetyl, NeuAc = *N*-acetylneuraminic acid, Nd = not determined.


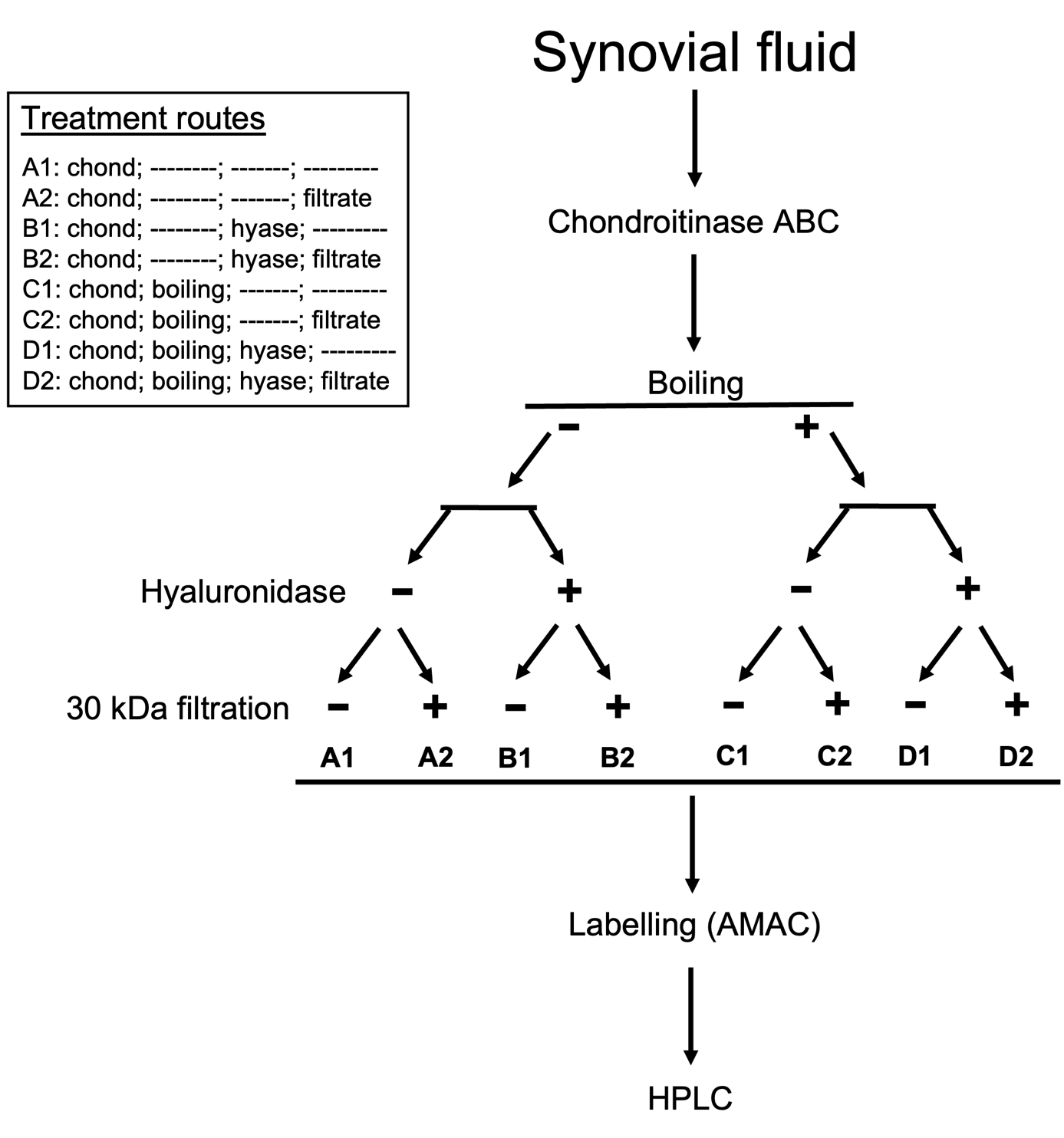


Figure S1. An overview of the different treatment strategies tested for synovial fluid samples before labelling and injection into high performance liquid chromatography (HPLC). The chondroitinase ABC digested synovial fluid samples were treated with different combinations of boiling, hyaluronidase digestion and 30 kDa filtration. AMAC = 2-aminoacridone, chond = chondroitinase ABC, hyase = hyaluronidase. For further details, see “Material and methods” section of the paper.


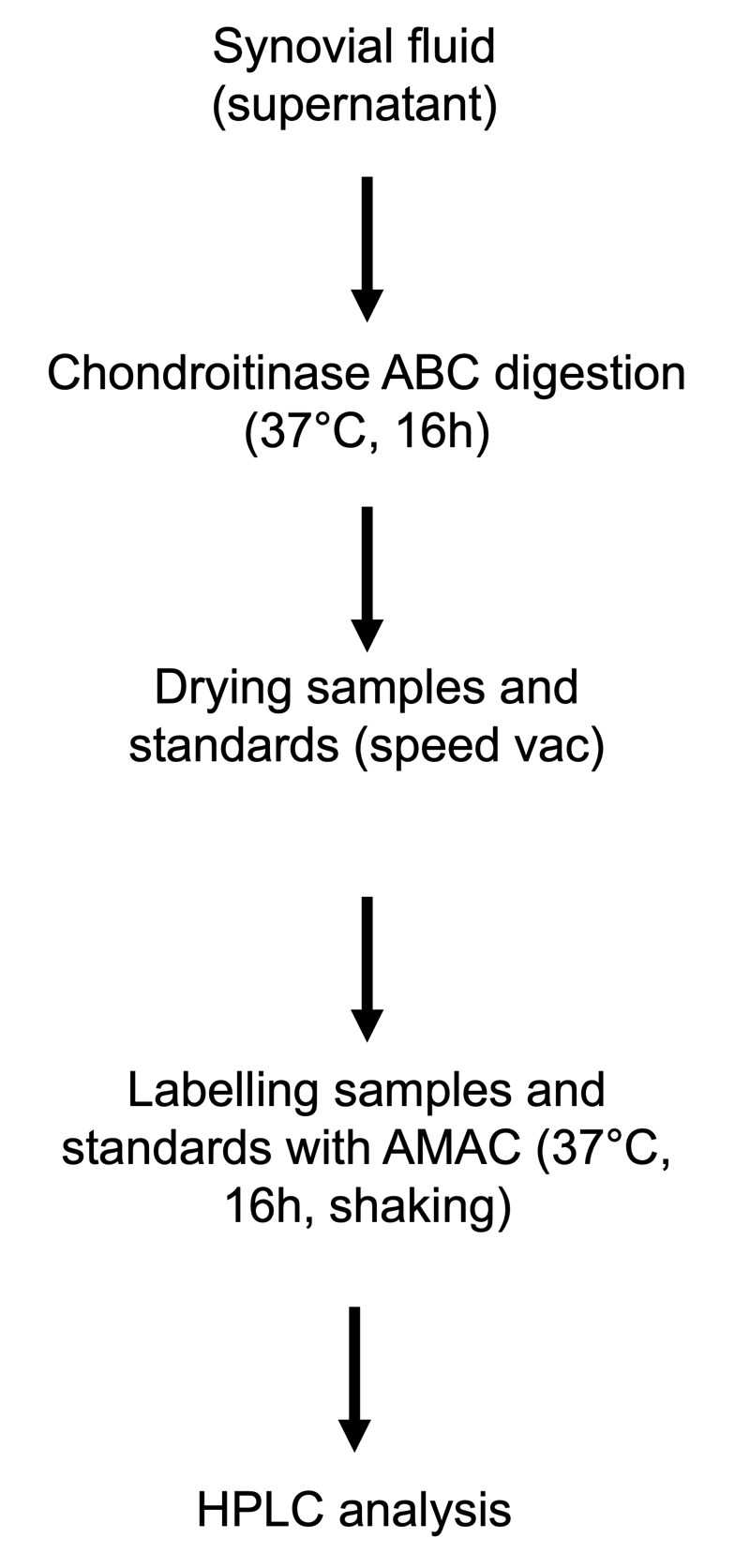


Figure S2. Schematic illustration of the workflow of the sample preparation before HPLC analysis. The method is described in more detail in the “Material and Methods” section of the paper. AMAC = 2-aminoacridone, HPLC = high performance liquid chromatography.


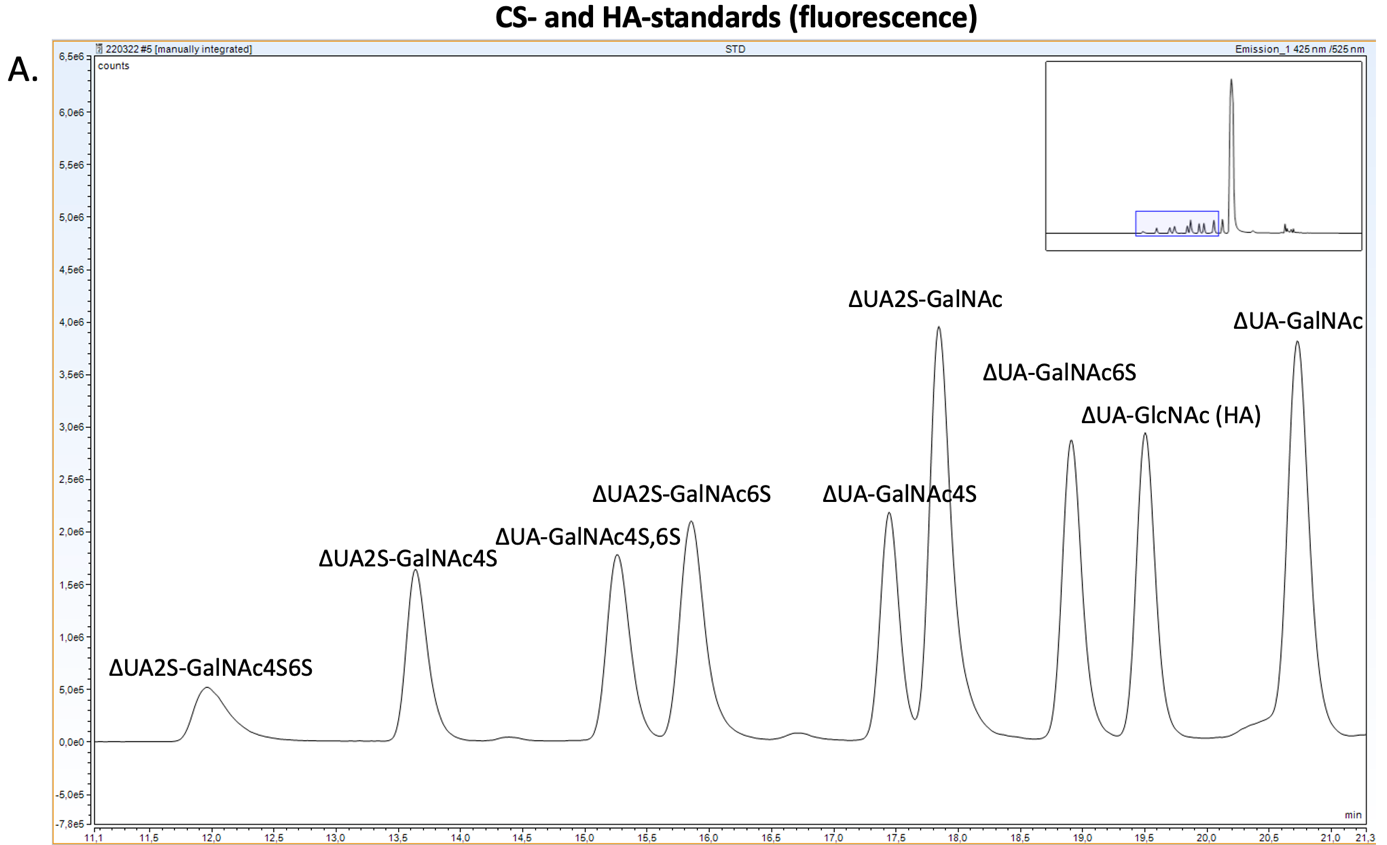


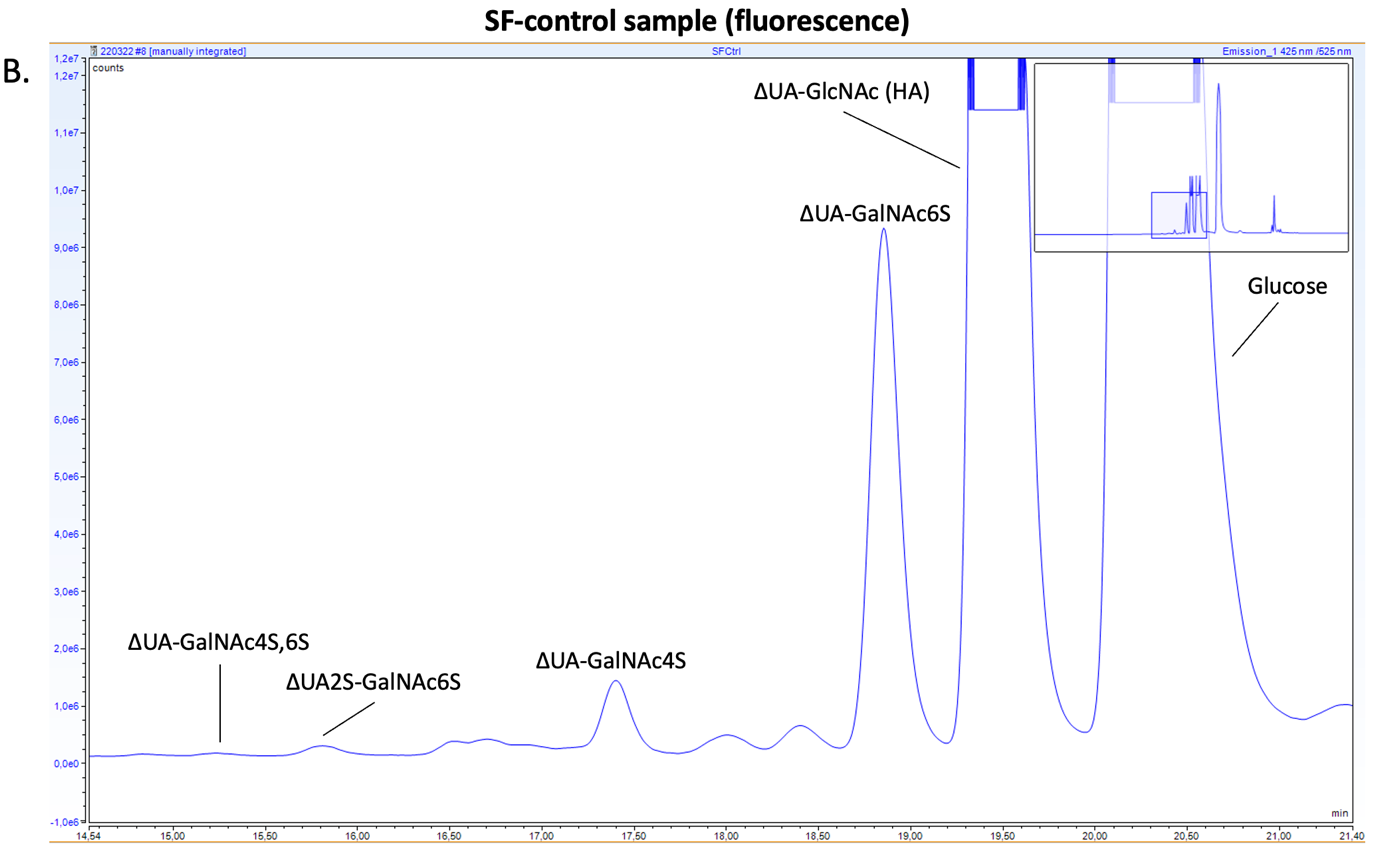


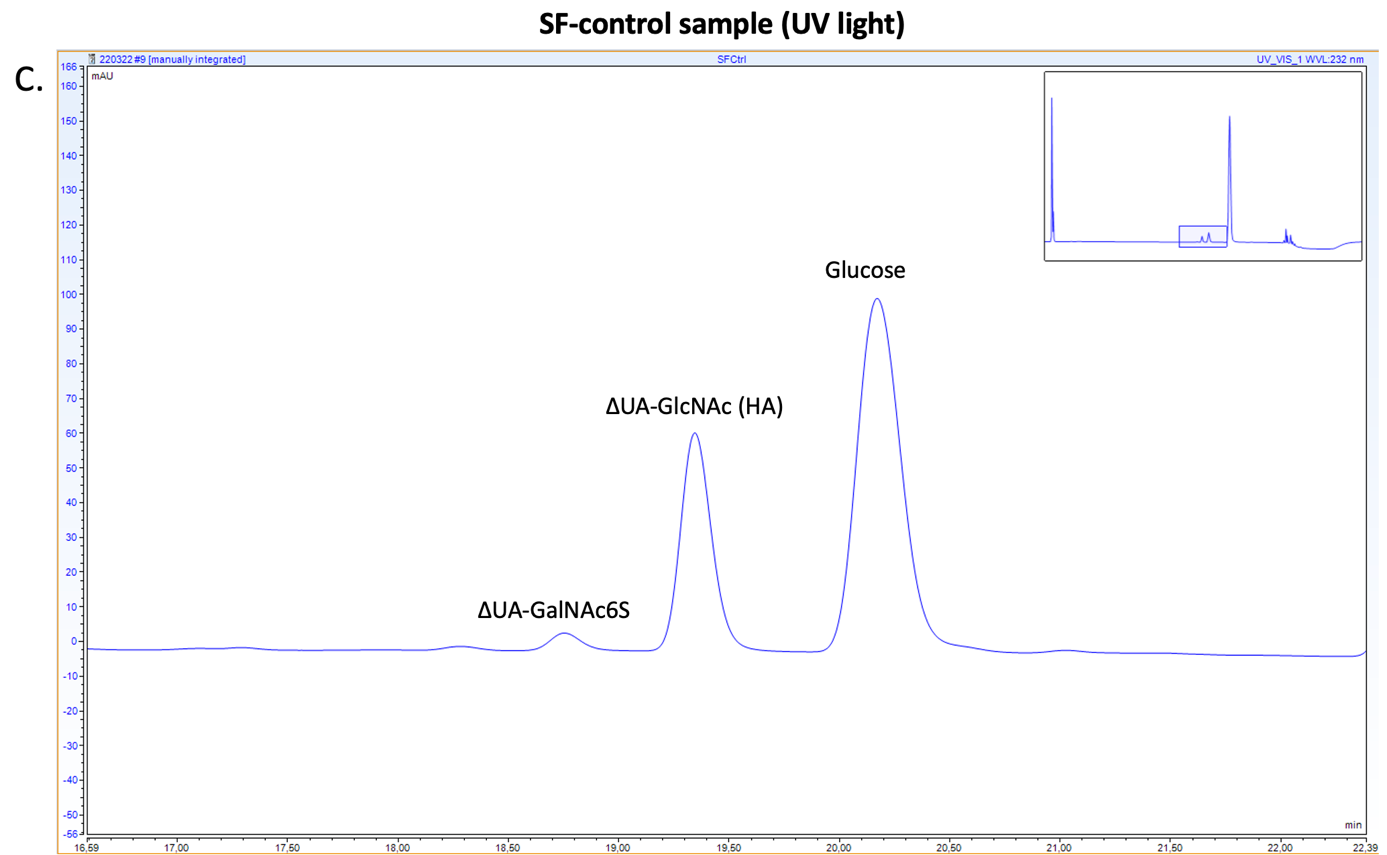


Figure S3. Elution profiles of unsaturated disaccharides derived from HA and CS. Panel-A, Standards (fluorescence). Panel-B, SF-control sample (fluorescence). Panel-C, SF-control sample (UV). Diluted synovial fluid was digested with chondroitinase ABC, labelled with AMAC and analyzed by HPLC. The CS-profiles were monitored by fluorescence detection (excitation λ = 425 and emission λ = 525) and HA either by fluorescence- or UV (232 nm) detection; the standards and samples were manually integrated. X-axis shows the retention time in minutes. SF-control sample is a synovial fluid pool from seven knee-injured patients. HA = hyaluronic acid, CS = chondroitin sulfate, AMAC = 2-aminoacridone HPLC = high performance liquid chromatography, GalNAc = *N*-acetylgalactosamine, GlcNAc = *N*-acetylglucosamine, ∆UA = unsaturated uronic acid, SF = synovial fluid. 2S, 4S and 6S are positions of sulfation.

**SUPPLEMENTARY REFERENCES**

S1. Jensen PH, Karlsson NG, Kolarich D, Packer NH. Structural analysis of N- and O-glycans released from glycoproteins. Nat Protoc 2012; 7: 1299-1310.

S2. Everest-Dass AV, Abrahams JL, Kolarich D, Packer NH, Campbell MP. Structural feature ions for distinguishing N- and O-linked glycan isomers by LC-ESI-IT MS/MS. J Am Soc Mass Spectrom 2013; 24: 895-906.

S3. Liu T, Qian WJ, Gritsenko MA, Camp DG, 2nd, Monroe ME, Moore RJ, et al. Human plasma N-glycoproteome analysis by immunoaffinity subtraction, hydrazide chemistry, and mass spectrometry. J Proteome Res 2005; 4: 2070-2080.
